# Flexible Cellular Phenotypes Retain a Shared Type-Level Organization across Response Regimes

**DOI:** 10.1523/ENEURO.0275-26.2026

**Published:** 2026-11-02

**Authors:** Zihan Yang

**Affiliations:** Department of Neuroscience, Yale University, New Haven, Connecticut 06510

**Keywords:** electrophysiological phenotype, Patch-seq, stimulus protocol dependence, transcriptomic cell types

## Abstract

Electrophysiological phenotype is widely used to characterize neuronal identity, but the phenotype measured from a neuron depends on how its intrinsic physiology is probed. Here, we asked which aspects of cellular electrophysiological organization persist across distinct response regimes and how this persistence relates to transcriptomic identity. Using matched Patch-seq measurements from visual cortical GABAergic neurons in male and female mice, we constructed phenotype spaces independently from long-pulse, ramp, and brief-pulse current-clamp responses and compared their organization across the same cells. Cell-level phenotypic relationships were only partially conserved across regimes: cell–cell phenotypic distances were positively correlated across regimes but far from identity, and local neighborhoods reorganized even where global rank structure was partly conserved. Despite this flexibility, the transcriptomic type consistently anchored phenotype beyond coarse Cre-driver identity. At the type level, phenotypic relationships were markedly more conserved across regimes, forming a shared relational backbone with a smaller set of regime-selective rewrites. Transcriptomic distance preferentially tracked this shared type-level structure rather than the magnitude of regime-selective reorganization, and predefined excitability-related gene programs showed the same asymmetry. Together, these results reveal a scale-dependent stability of neuronal phenotype: individual cellular relationships are flexible across probes, whereas type-level biological organization is comparatively stable and more strongly reflected in transcriptomic structure.

## Significance Statement

Electrophysiological properties are often treated as characteristics of neuronal identity, but the phenotype measured from a neuron depends on how that neuron is stimulated. By comparing multiple current-clamp response regimes in the same Patch-seq cells, we show that individual cellular relationships can reorganize substantially while relationships among transcriptomic cell types remain comparatively stable. Transcriptomic similarity preferentially corresponds to this shared type-level structure rather than to regime-dependent changes. These results suggest that cellular phenotype contains both a flexible, probe-dependent expression and a more stable biological organization, clarifying how electrophysiological and transcriptomic descriptions of neuronal identity relate across measurement conditions.

## Introduction

Neuronal identity is expressed through multiple levels of biological organization, from gene expression to the intrinsic electrical properties that determine how a cell transforms input into output. Electrophysiological properties—including membrane excitability, action-potential waveform, and repetitive firing—have therefore long provided an important description of neuronal phenotype and cellular diversity (Petilla Interneuron Nomenclature Group, 2008; Druckmann et al., 2013; Tremblay et al., 2016; Zeng and Sanes, 2017). Yet electrophysiological phenotype is necessarily observed through the conditions used to probe it. Different current-clamp stimuli recruit different components of intrinsic physiology, raising a basic question about cellular phenotype: which aspects of electrophysiological organization persist when the same cells are probed under different response regimes?

Single-cell transcriptomics and Patch-seq have provided a molecular framework in which to address this problem. Transcriptomic studies have resolved extensive neuronal diversity in the mammalian cortex (Zeisel et al., 2015; Tasic et al., 2016, 2018; Hodge et al., 2019), while Patch-seq makes it possible to measure transcriptomic identity and intrinsic electrophysiological properties in the same cells (Cadwell et al., 2016; Fuzik et al., 2016; Lee et al., 2021; Lipovsek et al., 2021). Large-scale multimodal studies have demonstrated substantial correspondence between transcriptomic types and electrophysiological or morphoelectric phenotypes (Gouwens et al., 2019, 2020; Scala et al., 2021), and earlier cross-dataset analyses likewise identified systematic relationships between gene-expression patterns and intrinsic electrophysiological properties (Tripathy et al., 2017; Bomkamp et al., 2019). These findings establish that molecular identity is reflected in cellular physiology, but they do not imply a one-to-one mapping between molecular class and every aspect of electrophysiological phenotype. Indeed, Patch-seq was developed in part to quantify correspondence across modalities while retaining the biological variability present within neuronal populations.

A remaining question is how this molecular–electrophysiological correspondence is organized across different ways of probing cellular response. Electrophysiological measurements obtained under different stimulus regimes could simply resample a common underlying organization, in which case relative relationships among cells and cell types should remain largely conserved. Alternatively, different regimes could reveal distinct components of cellular physiology, reorganizing individual-cell relationships while preserving only a coarser biological structure. Importantly, these possibilities separate two levels of phenotypic organization that are often treated together: the arrangement of individual cells and the relationships among biological cell types. A cell may change its phenotypic position across response regimes without erasing the type-level organization in which it is embedded.

This distinction also provides a way to ask what molecular organization explains. If transcriptomic identity specifies a common electrophysiological backbone, molecular similarity should preferentially correspond to phenotypic relationships that persist across response regimes. If molecular differences instead determine regime-selective expression, they should also predict which phenotypic relationships are reorganized by different stimuli. Separating shared from regime-selective structure therefore allows molecular correspondence to be evaluated against distinct components of cellular phenotype rather than against a single aggregate electrophysiological representation.

Here, we reanalyzed the mouse visual cortex GABAergic interneuron Patch-seq dataset generated by Gouwens et al. (2020), in which electrophysiological and transcriptomic measurements were obtained from the same cells across long-pulse, ramp, and brief-pulse current-clamp regimes. Because this dataset provides matched electrophysiological and transcriptomic measurements at single-cell resolution, it allows phenotypic organization to be defined independently within each regime before transcriptomic identity is introduced as an explanatory layer. We first asked how cellular relationships were conserved or reorganized across response regimes. We then tested whether these organizations remained anchored by transcriptomic type beyond coarse Cre-driver identity and whether relationships among transcriptomic types formed a common structure across regimes. Finally, we asked whether transcriptomic similarity and prespecified molecular programs preferentially corresponded to shared or regime-selective components of this type-level organization.

We find that cellular electrophysiological phenotypes are partially conserved across response regimes but undergo substantial reorganization at the level of local cellular neighborhoods, across a range of neighborhood scales. Despite this flexibility, the transcriptomic type consistently anchors phenotype beyond Cre identity, and relationships among transcriptomic types form a broadly shared backbone with a smaller set of regime-selective rewrites. Transcriptomic similarity and predefined excitability-related programs preferentially correspond to the shared component rather than to the extent of selective rewriting. Together, these results reveal a scale-dependent stability of cellular phenotype: individual-cell relationships are flexible across response regimes, whereas type-level organization is comparatively stable and preferentially reflected in transcriptomic structure.

## Materials and Methods

### Dataset and animals

We reanalyzed publicly released Patch-seq recordings from mouse visual cortex GABAergic neurons, predominantly the primary visual cortex (VISp) with a minority of cells from neighboring visual areas (Gouwens et al., 2020; Allen Institute for Brain Science). This study is a reanalysis of previously collected data; no new animals were used. Current-clamp electrophysiology was obtained from DANDI dandiset 000020, version 0.210913.1639 (DOI: 10.48324/dandi.000020/0.210913.1639; 4,435 files with the suffix _icephys.nwb). Matched transcriptomic metadata and gene-count tables were obtained from the Neuroscience Multi-omic Archive (NeMO) Allen Institute Patch-seq release (20200611; count matrix v2).

According to the original study, recordings were performed in male and female C57BL/6J-background mice [approximately postnatal days (P)45–70] carrying Cre-driver lines that enrich for cortical interneurons (Taniguchi et al., 2011). Animals were group-housed (≤5 mice per cage) with food and water available *ad libitum* on a regular 12 h light/dark cycle, and all procedures generating the source dataset were approved by the Institutional Animal Care and Use Committee at the Allen Institute for Brain Science (Gouwens et al., 2020); the present study involved no new animal procedures. Slice preparation, recording temperature, synaptic blockers, and SMART-Seq v4 library construction followed the published Patch-seq pipeline; the tissue was not recollected for this study.

Inclusion required successful feature extraction with the Allen Institute Intrinsic Physiology Feature Extractor (IPFX; extract_ok = 1). Analyses using transcriptomic labels further required a mapped transcriptomic type (t-type) and/or Cre-line field in the joined metadata table (*n* = 4,430 cells with a join row; *n* = 4,266 with a non-empty t-type).

### Electrophysiological feature extraction

For each Neurodata Without Borders (NWB) file, cell-level intrinsic features were extracted using IPFX. Features were drawn from three stimulus families, treated as response regimes rather than competing assays: long-pulse (long squares; sustained firing), ramp (recruitment/rheobase-like responses), and brief-pulse (short squares; transient responses); example current-clamp traces from each regime are shown in Figure 1*A*. Empty epoch fields in NWB2 files were dropped so that IPFX could recover stimulus and experiment windows from the traces; only sweeps with a usable stimulus window contributed features. Feature counts and dataset sizes per regime are summarized in Table 1. Feature counts per regime are summarized in Extended Data Figure 1-1*A*; full feature lists are given in Extended Data Table 1-1.

**Table 1.** Regime-specific feature counts, cell counts, and retained principal components (*k*_80_)

Regime	Cells	Features	*k*_80_
Long-pulse	4,344	42	11
Ramp	4,139	20	3
Brief-pulse	4,422	21	5

Related to Materials and Methods. Extended Data Table 1-1 lists retained IPFX features. Extended Data Table 2-1 is the statistical inventory.

10.1523/ENEURO.0275-26.2026.t1-1 Table 1-1 **Features by protocol.** IPFX feature column names retained for long-pulse, ramp, and brief-pulse phenotype construction (missingness ≤30%, nonzero variance). Download Table 1-1, DOCX file.

### Protocol-resolved cellular phenotype

For each regime independently, numeric IPFX features with ≤30% missing values and nonzero variance were retained, remaining missing entries were imputed with the feature mean, columns were *z*-scored, and principal component analysis (PCA) was computed via singular value decomposition (SVD) of the standardized feature matrix. The phenotype of cell *i* in regime *p* was defined as its score vector $R_{i}^{p}$ in the first *k*_80_ principal components, where *k*_80_ is the smallest number of components explaining at least 80% of the variance. Resulting dataset sizes are summarized in Table 1 (long-pulse: 4,344 cells, 42 features, *k*_80_ = 11; ramp: 4,139 cells, 20 features, *k*_80_ = 3; brief-pulse: 4,422 cells, 21 features, *k*_80_ = 5).

PCA was computed independently in each regime. Feature loadings on PC1 (and the next components) were retained from the SVD of the *z*-scored feature matrix and are reported in Extended Data Figure 1-2*D*–*F*. Cross-regime comparisons in the main text use pairwise distances in the full *k*_80_ space rather than PC1 scores.

Molecular labels were not used to construct phenotype spaces. Pairwise phenotypic distance $D_{\mathrm{phenotype}}^{p}$ was defined as Euclidean distance in the *k*_80_ score space. Sensitivity analyses additionally retained a fixed five principal components in each regime (Extended Data Fig. 1-2). Same-cell overlaps for cross-regime comparisons were as follows: long-pulse ∩ ramp, *n* = 4,054; long-pulse ∩ brief-pulse, *n* = 4,336; ramp ∩ brief-pulse, *n* = 4,134; and triple overlap, *n* = 4,049.

### Molecular labels

The primary discrete label used for anchoring and type–type analyses was the published transcriptomic t-type; Cre/driver-line identity was used only as a control partition. Continuous transcriptomic geometry (see final Results subsection) used log-CPM normalized NeMO counts joined by transcriptomic sample identifier. Three gene panels were prespecified: a curated excitability list (263 genes); a primary panel of 500 genes, constructed as the union of the curated list and additional genes ranked by expression variance, padded to a total of 500; and an independently defined panel of the top 500 highly variable genes by expression variance alone (HVG500). Primary-panel gene selection used only the curated gene list and expression-variance ranking, was fixed before any phenotype or type–type association analysis, and did not incorporate t-type marker information or phenotypic results; it is termed “primary” because it combines curated and variance-ranked coverage, whereas the curated and HVG500 panels serve as independent single-criterion robustness checks. Excitability modules (Nav, Kv3, Kv delayed rectifier, HCN, Cav, SK/BK, potassium-leak, GABA_A_, AMPA/NMDA) and subclass marker controls (Pvalb, Sst, Vip) were defined before analysis for use in the transcriptomic-geometry analyses reported below.

### Analysis pipeline

Analyses followed a fixed order, moving from a label-agnostic description of phenotype (constructed without reference to transcriptomic labels), to biological anchoring, to comparison of type-level organization across regimes, and finally to a test of molecular explanation.

### Cross-regime phenotypic organization

For each pair of regimes, on the corresponding shared-cell set, we sampled up to 80,000 unique cell pairs (seed = 0), computed Euclidean distances in each regime's *k*_80_ space, and measured the Spearman correlation between the two distance vectors. Neighborhood conservation was quantified as the Jaccard overlap of each cell's *k* nearest neighbors in the two regime spaces, computed for *k* = 15, 30, 60, 120, 240, 480, and 600. Because expected overlap increases with *k* under random pairing, we compared the observed mean Jaccard with the mean Jaccard after 100 index shuffles of cell pairing (seed = 0) and report the chance-adjusted difference (observed − chance) at each *k* (Extended Data Fig. 1-2). Null distributions were generated by shuffling cell indices within one regime (*n* = 100 permutations); empirical *p* values were computed as (1 + number of null values ≥ observed)/(1 + number of permutations).

For cells with at least four usable long-pulse sweeps, current-clamp sweeps from the archived DANDI 000020 NWB files were split into two halves (each half required ≥2 sweeps) using a fixed candidate order: odd/even by sweep number, interleaved by sweep-number rank, interleaved by stimulus-amplitude rank (when amplitude metadata was available), and random (seed = 0). IPFX long-pulse feature extraction was attempted on each candidate split in this order, and the first candidate for which extraction succeeded on both halves was retained (of the 3,598 cells with a successful split: odd/even, *n* = 3,112; sweep-number interleaved, *n* = 128; amplitude-interleaved, *n* = 0; random, *n* = 358). Retained-half features were *z*-scored using the mean and standard deviation of the full-cell long-pulse feature table and projected onto the original PCA loadings (*k*_80_ = 11 components); half-split labels did not enter that fit. Neighborhood reliability was quantified as the 15-nearest-neighbor Jaccard overlap between half-A and half-B phenotype positions for the 3,598 cells with successful extraction from both halves, compared with cross-regime 15-NN Jaccard recomputed in the corresponding cell pools (long–ramp *n* = 3,276; long–brief *n* = 3,509; ramp–brief *n* = 3,351; chance from 100 index shuffles; Extended Data Fig. 1-3). Ramp and brief-pulse responses had insufficient repeated sweeps for the same analysis.

### Biological anchoring

Biological anchoring across t-type and Cre identity is summarized in Figure 2 (multivariate *η*^2^ and same-type neighbor enrichment) and Extended Data Figure 2-1 (pairwise-distance alignment statistics). For cells with an assigned t-type (and, separately, Cre-line identity), the primary alignment statistic A^p^ was the Spearman correlation between label distance *D*_mol_ and phenotypic distance $D_{\mathrm{phenotype}}^{p}$, computed on up to 80,000 pairs; *D*_mol_ was defined as binary mismatch for discrete labels, with hierarchical subtype distance used where reported; we denote this statistic *A_t_* when the discrete label is t-type. Multivariate *η*^2^ (reported in Fig. 2*C*) was the fraction of total sum of squares in *k*_80_ phenotype space associated with a discrete label: if *X* is the *n* × *k* score matrix, $\bar{x}$ is the grand mean, and ${\bar{x}}_{g}$ is the mean of group *g* with size *n_g_*, $\eta^{2}=\sum_{g}n_{g}\|{\bar{x}}_{g}-\bar{x}{\|}^{2}/\sum_{i}\|x_{i}-\bar{x}{\|}^{2}$. This is the multivariate analog of between-group sum of squares over total sum of squares. Cre identity was assessed on the same cells that had a Cre label. Residual t-type structure after accounting for Cre identity was assessed by subtracting, within each Cre line, that line's mean score vector, then recomputing *η*^2^ (and *A_t_*) for t-type on the residualized scores. Same-type nearest-neighbor enrichment (Fig. 2*D*) was defined as the fraction of each cell's 15 nearest neighbors sharing its t-type, divided by chance, where chance for cell *i* was (*n*_type(*i*)_ − 1)/(*n* − 1). Label-shuffle null distributions used 100 permutations (seed = 0).

### Type–type relationships across regimes

For each regime, methods underlying the type–type relational maps reported in Figure 3 were as follows. t-type centroids were computed in *k*_80_ phenotype space for the 57 t-types represented by at least 10 cells in all three regimes (Extended Data Fig. 3-1), yielding 1,596 unordered type pairs. Type–type phenotypic distance *S_p_* (a,b) was defined as the Euclidean distance between the centroids of types a and b. Concordance between regimes was quantified as the Spearman correlation of the corresponding upper-triangle distance vectors, with type-label shuffle nulls (*n* = 1,000). A prespecified model call classified the overall pattern (shared, partial, or regime-specific) using the three pairwise concordance values, with a shared-map call requiring a minimum pairwise *ρ* ≥ 0.40 and a range across the three concordances <0.25. Individual type pairs were tagged, based on rank-normalized *S* values across the three regimes, as stable (rank coefficient of variation <0.25), regime-selective (rank range ≥0.55), or intermediate; threshold sensitivity is reported in Extended Data Figure 3-2. Subclass-level concordance used the six subclasses shared across regimes. To assess whether concordance was inflated by unequal per-type sampling, centroids were additionally rebuilt from equal-N draws (10 cells per type per regime; 200 resamples, seed = 0), and within-regime split-half *S* concordance (200 resamples) was computed as a sampling-reliability ceiling for comparison (Extended Data Fig. 3-3).

### Molecular recovery of shared versus regime-selective structure

Type–type transcriptomic distance *T*(a, b) was defined as the Euclidean distance between *t*-type centroids in the first 10 principal components of each gene panel. We compared *T* with two properties of the phenotypic type map: cross-regime mean phenotypic separation—the mean of *S_p_* (a,b) across the three regimes, capturing the component of type-level separation expressed on average across regimes—and the cross-regime rank range of rank-normalized *S_p_* (a, b; max minus min rank across regimes), quantifying regime-selective reorganization. Label-shuffle empirical *p* values used 1,000 permutations for the primary 500-gene panel and 50 permutations for the curated, HVG500, and module-restricted panels. Module-level tests repeated the same contrasts using module-restricted transcriptomic distances for the prespecified excitability modules (Fig. 4*C*; module gene sets listed in Extended Data Fig. 4-1) and for the subclass marker controls (Extended Data Fig. 4-2*C*).

### Transcriptomic mobility and coexpression analysis

Mobility for cell *i* was defined as one minus the mean Spearman correlation of *i*'s vector of Euclidean distances to all other triple-shared cells, compared across the three pairs of *k*_80_ regime spaces (self-comparisons excluded). NeMO Patch-seq counts were log-CPM normalized and joined by transcriptomic sample identifier, as in the type–type transcriptomic analyses above; of the 4,049 triple-shared cells, 3,906 had a matched transcriptomic sample identifier and an assigned transcriptomic type and were retained for network construction and the unadjusted module–mobility correlation. Genes were the union of the prespecified excitability list and highly variable genes, capped at 2,000 total (263 curated genes present). Weighted gene coexpression network analysis (WGCNA; Langfelder and Horvath, 2008; v1.74) was used to construct coexpression modules on this panel: an unsigned network was built following goodSamplesGenes quality control, with the soft-threshold power β chosen as the lowest power reaching a scale-free topology fit *R*^2^ ≥ 0.8 (*β* = 3, *R*^2^ = 0.891), and modules were identified with blockwiseModules (minimum module size = 20, merge cut height = 0.25). The correlation between each module eigengene and mobility was assessed with Spearman's *ρ*, with empirical *p* values from 1,000 label shuffles, computed as (1 + count)/(1 + 1,000). To test whether these associations reflected within-type variation rather than transcriptomic type identity, mobility and each module eigengene were mean-centered within the transcriptomic type before recomputing the correlation, restricted to types with at least three members (*n* = 3,902). Gray (unassigned) genes were not treated as a module.

### Statistics

All analyses were descriptive and estimation-oriented, applied to an archival observational cohort with a nonrandom sampling design. Primary effect sizes were Spearman's *ρ*, *η*^2^, nearest-neighbor enrichment ratios, and pair-classification proportions; uncertainty for key correlations was conveyed by permutation null distributions and empirical *p* values, computed as (1 + number of null values ≥ observed)/(1 + number of permutations). Specific *p* values are reported throughout rather than inequalities. Sample sizes, effect sizes, and *p* values for reported contrasts are compiled in Table 2 (rows a–y; see also end of Results). Null statistics were retained, and descriptive effect sizes without a permutation *p* value (e.g., *η*^2^, nearest-neighbor enrichment) are in Extended Data Table 2-1. Random seeds were fixed (seed = 0) in all analysis scripts.

**Table 2.** Statistical summary of reported contrasts

Letter	Data structure	Type of test	Statistical results
a	D, long–ramp, *n* = 4,054	Spearman (100)	*ρ* = 0.434, *p* = 0.0099
b	D, ramp–brief, *n* = 4,134	Spearman (100)	*ρ* = 0.384, *p* = 0.0099
c	D, long–brief, *n* = 4,336	Spearman (100)	*ρ* = 0.336, *p* = 0.0099
d	*t*-type, long, *n* = 4,183	Spearman *A_t_* (100)	*A_t_* = 0.021, *p* = 0.0099
e	*t*-type, ramp, *n* = 3,988	Spearman *A_t_* (100)	*A_t_* = 0.025, *p* = 0.0099
f	*t*-type, brief, *n* = 4,266	Spearman *A_t_* (100)	*A_t_* = 0.021, *p* = 0.0099
g	After Cre, long, *n* = 4,183	Spearman *A_t_* (100)	*A_t_* = 0.011, *p* = 0.0099
h	After Cre, ramp, *n* = 3,988	Spearman *A_t_* (100)	*A_t_* = 0.010, *p* = 0.0099
i	After Cre, brief, *n* = 4,266	Spearman *A_t_* (100)	*A_t_* = 0.004, *p* = 0.0099
j	S, long–ramp, *n* = 1,596	Spearman (1,000)	*ρ* = 0.658, *p* = 0.000999
k	S, ramp–brief, *n* = 1,596	Spearman (1,000)	*ρ* = 0.608, *p* = 0.000999
l	S, long–brief, *n* = 1,596	Spearman (1,000)	*ρ* = 0.455, *p* = 0.000999
m	Primary *T* versus mean *S*	Spearman (1,000)	*ρ* = 0.507, *p* = 0.000999
n	Primary *T* versus rank range	Spearman (1,000)	*ρ* = 0.083, *p* = 0.000999
o	Curated *T* versus mean *S*	Spearman (50)	*ρ* = 0.551, *p* = 0.020
p	HVG500 versus mean S	Spearman (50)	*ρ* = 0.520, *p* = 0.020
q	Curated *T* versus rank range	Spearman (50)	*ρ* = 0.011, *p* = 0.706
r	HVG500 versus rank range	Spearman (50)	*ρ* = 0.105, *p* = 0.020
s	Modules, *n* = 1,596	*ρ* versus mean S (50)	*ρ* = 0.314, *p* = 0.020
t	Modules, *n* = 1,596	*ρ* versus range (50)	*ρ* = −0.023, *p* = 0.294
u	Split-half, *n* = 3,598	Jaccard (100)	adj. = 0.107 (obs. = 0.109)
v	Split-half, 3 pairs	Jaccard (100)	0.025/0.024/0.020
w	kNN, *k* = 15–600	Obs—chance (100)	0.134/0.101/0.108
x	Mobility, *n* = 4,049	1—mean ρ	0.589 ± 0.110
y	WGCNA, *n* = 3,902	Spearman (1,000)	|*ρ*| ≤ 0.039

Letters match superscripts in Results. Full inventory: Extended Data Table 2-1.

10.1523/ENEURO.0275-26.2026.t2-1 Table 2-1 **Statistical inventory.** Master list of reported contrasts: question, test, n, effect size, null mean, empirical p, and notes. Null means are listed where they were retained; descriptive η² / kNN / tag counts have no shuffle p. Download Table 2-1, DOCX file.

### Code accessibility

The code/software described in the paper is freely available online at https://github.com/zy-neuro/protocol-response-geometry. The code is available as Extended Data. Analyses were run on an Apple Silicon workstation (macOS) using Python 3 with NumPy and Matplotlib and the Allen Institute IPFX package, within a dedicated project virtual environment. WGCNA used the WGCNA R package (v1.74) in addition to the Python-based analysis pipeline.

10.1523/ENEURO.0275-26.2026.d1 Data 1 Analysis scripts, configuration files, and figure-generation code used to produce the results in this paper. See Materials and Methods, Code Accessibility. Download Data 1, ZIP file.

### Software accessibility

The analysis and modeling software was custom-written for this study. The software is freely available online at https://github.com/zy-neuro/protocol-response-geometry. If it is not deposited with the article, it will be provided to other scientists upon request.

### Data accessibility

Electrophysiology data are available from DANDI dandiset 000020 (https://dandiarchive.org/dandiset/000020/0.210913.1639). Transcriptomic data are available from the NeMO Allen Institute Patch-seq release (20200611). Derived phenotype tables, metadata joins, and summary tables sufficient to regenerate all figures are included with the code (see above, Code accessibility).

## Results

### Cellular phenotypes are partially conserved and locally reorganized across response regimes

To examine how cellular electrophysiological phenotype depends on the response regime in which it is measured, we analyzed three current-clamp stimulus regimes from the Patch-seq dataset: long-pulse, ramp, and brief-pulse stimulation. Electrophysiological features were extracted independently for each regime using IPFX, standardized across cells, and represented using PCA, with components retained to explain 80% of the variance. Long-pulse responses comprised 42 features across 4,344 cells and required 11 principal components to reach this threshold; ramp responses comprised 20 features across 4,139 cells and required three components; and brief-pulse responses comprised 21 features across 4,422 cells and required five components (Table 1, Extended Data Fig. 1-1*A*). Because principal components were fit independently in each regime, we do not treat PC1 as a shared biological axis across regimes; loadings of the leading features in each regime are reported in Extended Data Figure 1-2*D–F*. Thus, each response regime provided a structured but differently dimensional view of cellular electrophysiological phenotype.

Pairwise Euclidean distances in the retained phenotype spaces were positively correlated across all three regime pairs, above shuffled-null expectations (empirical *p* = 0.0099 for each), although substantially below identity. Conservation was strongest between long-pulse and ramp responses (Spearman's *ρ* = 0.434^a^, *n* = 4,054 shared cells), followed by ramp and brief-pulse responses (*ρ* = 0.384^b^, *n* = 4,134) and long-pulse and brief-pulse responses (*ρ* = 0.336^c^, *n* = 4,336; Fig. 1*B*); this ordering was unchanged when a fixed number of five principal components was retained instead of the 80% variance threshold (Extended Data Fig. 1-2*A*).

**Figure 1.**
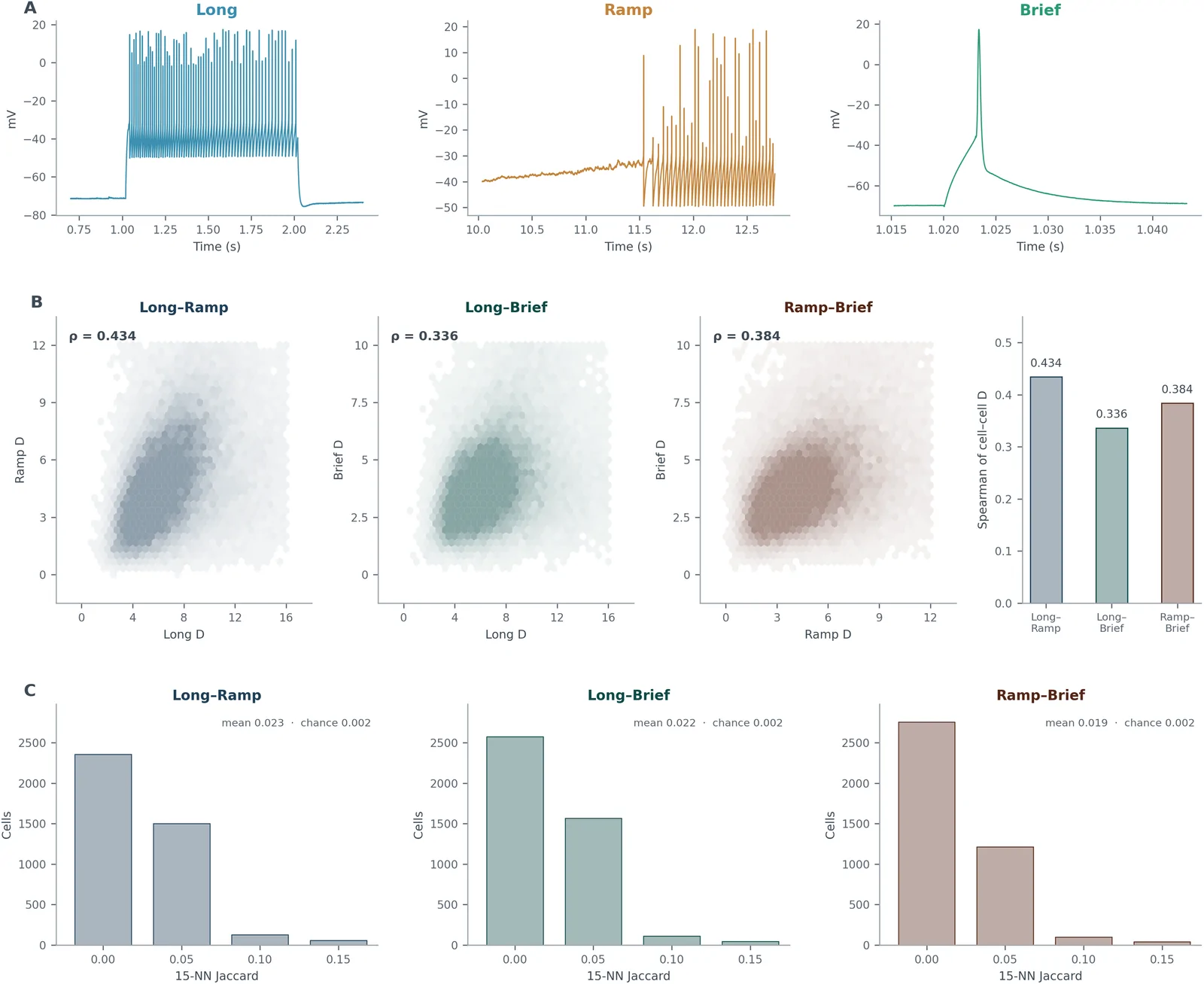
Cellular phenotype across response regimes. ***A***, Example current-clamp voltage traces from the three regimes (long-pulse, ramp, brief-pulse) for the same cell (DANDI session 639797293). Scale bar, time (s), membrane potential (mV). ***B***, Global organization: hexbin of pairwise Euclidean distances D in *k*_80_ phenotype space for shared cells (*ρ* on each panel), and Spearman correlation of those distances (long–ramp *n* = 4,054; long–brief *n* = 4,336; ramp–brief *n* = 4,134); empirical *p* = 0.0099 versus index shuffle for each pair. ***C***, Local organization: distribution of Jaccard overlap of each cell's 15 nearest neighbors across the same three pairs; chance is the mean Jaccard under index shuffle (≈0.002). Protocol colors are long-pulse, blue; ramp, apricot; and brief-pulse, green. Cross-regime pairs use a separate muted palette: slate (Long–Ramp), teal (Long–Brief), sienna (Ramp–Brief). See also Extended Data Figures 1-1–1-3.

10.1523/ENEURO.0275-26.2026.f1-1 Figure 1-1 **Phenotype construction. A**, Number of retained IPFX features and k_80_ principal components per regime; n cells under each protocol label. **B**, Cumulative variance explained versus PC index; dashed line marks 80%; markers indicate k_80_. **C**, Per-component variance fraction (scree) for the first 15 PCs. Download Figure 1-1, TIF file.

10.1523/ENEURO.0275-26.2026.f1-2 Figure 1-2 **Cross-regime robustness and PC1 loadings. A**, Spearman of cell–cell D for k_80_ (open, pair-colored edge) versus the fixed 5-PC representation (filled, pair-colored) on the same shared-cell pairs. **B**, Mean k-nearest-neighbor (kNN) Jaccard overlap as a function of neighborhood size (*k* = 15–600; color and marker encode the protocol pair), with the mean under 100 index shuffles of cell pairing shown as a dotted line and band (range across pairs). **C**, Chance-adjusted Jaccard (observed − chance) versus k for the same neighborhood-size sweep. **D–F**, Leading PC1 feature loadings for long-pulse, ramp, and brief-pulse, respectively (independent PCAs; features ranked by absolute loading). Download Figure 1-2, TIF file.

10.1523/ENEURO.0275-26.2026.f1-3 Figure 1-3 **Within-regime split-half versus cross-regime neighborhood overlap. A**, 15-nearest-neighbor Jaccard overlap between half-A and half-B (ink fill; the within-regime control) compared with each cross-regime pair on the same cells. **B**, Mean 15-NN Jaccard for the within-regime split-half and the three cross-regime pairs on the same cells; *n* = 3,598 cells with both halves. Download Figure 1-3, TIF file.

Local cellular organization was less stable still: mean 15-nearest-neighbor Jaccard overlap was 0.023, 0.022, and 0.019 for the long-pulse–ramp, long-pulse–brief-pulse, and ramp–brief-pulse pairs, only modestly above index-shuffle chance (≈0.002; Fig. 1*C*), indicating extensive local reorganization despite the positive correspondence in global pairwise-distance ranks. Repeating this comparison across a broader range of neighborhood sizes (*k* = 15, 30, 60, 120, 240, 480, and 600) showed that chance-adjusted overlap increased with neighborhood size but remained low even at *k* = 600 (0.134, 0.101, and 0.108 for the same three pairs^w^), indicating that local reorganization is not confined to the smallest neighborhoods (Extended Data Fig. 1-2*B*,*C*).

To assess whether trial-to-trial variability could account for the low cross-regime overlap, we split usable long-pulse sweeps into disjoint halves, extracted IPFX long-pulse features independently on each half, and projected both halves into the original long-pulse phenotype space (full-cell *z*-score and PCA; half-split labels did not enter that fit). On the 3,598 cells with both halves successfully extracted, mean 15-NN Jaccard overlap between half-A and half-B was 0.109 (chance ≈  0.0026; chance-adjusted 0.107^u^), compared with 0.025, 0.024, and 0.020^v^ for the long-pulse–ramp, long-pulse–brief-pulse, and ramp–brief-pulse pairs recomputed in that pool (*n* = 3,276/3,509/3,351; Extended Data Fig. 1-3). Within-regime neighborhood reliability was therefore substantially higher than cross-regime overlap, although split-half overlap remained far from complete, indicating that trial-level variability contributes to, but does not fully account for, the cross-regime deficit.

Together, these results indicate that cellular electrophysiological phenotype is partially conserved across response regimes but substantially reorganized at the level of local neighborhoods—a pattern that persists across neighborhood scales and cannot be accounted for by within-regime trial variability alone, with the strongest overall conservation between long-pulse and ramp responses.

### Transcriptomic type anchors cellular phenotype beyond coarse Cre-driver identity

The partial preservation of phenotypic structure across response regimes raised the question of whether these cellular organizations remained anchored to biological identity. We therefore introduced transcriptomic labels only after constructing the phenotype spaces. Among cells with mapped transcriptomic types (t-types; *n* = 4,183 for long-pulse, 3,988 for ramp, and 4,266 for brief-pulse), transcriptomic identities occupied distinct but overlapping portions of phenotype space, and finer t-type structure remained apparent within individual Cre-driver populations (Fig. 2*A*, subclass occupancy; Fig. 2*B*, t-type structure within the Sst-IRES-Cre driver). Long-pulse PC1, used here only as a within-regime axis, loads most strongly on rheobase/threshold current and input resistance (Extended Data Fig. 1-2*D*).

**Figure 2.**
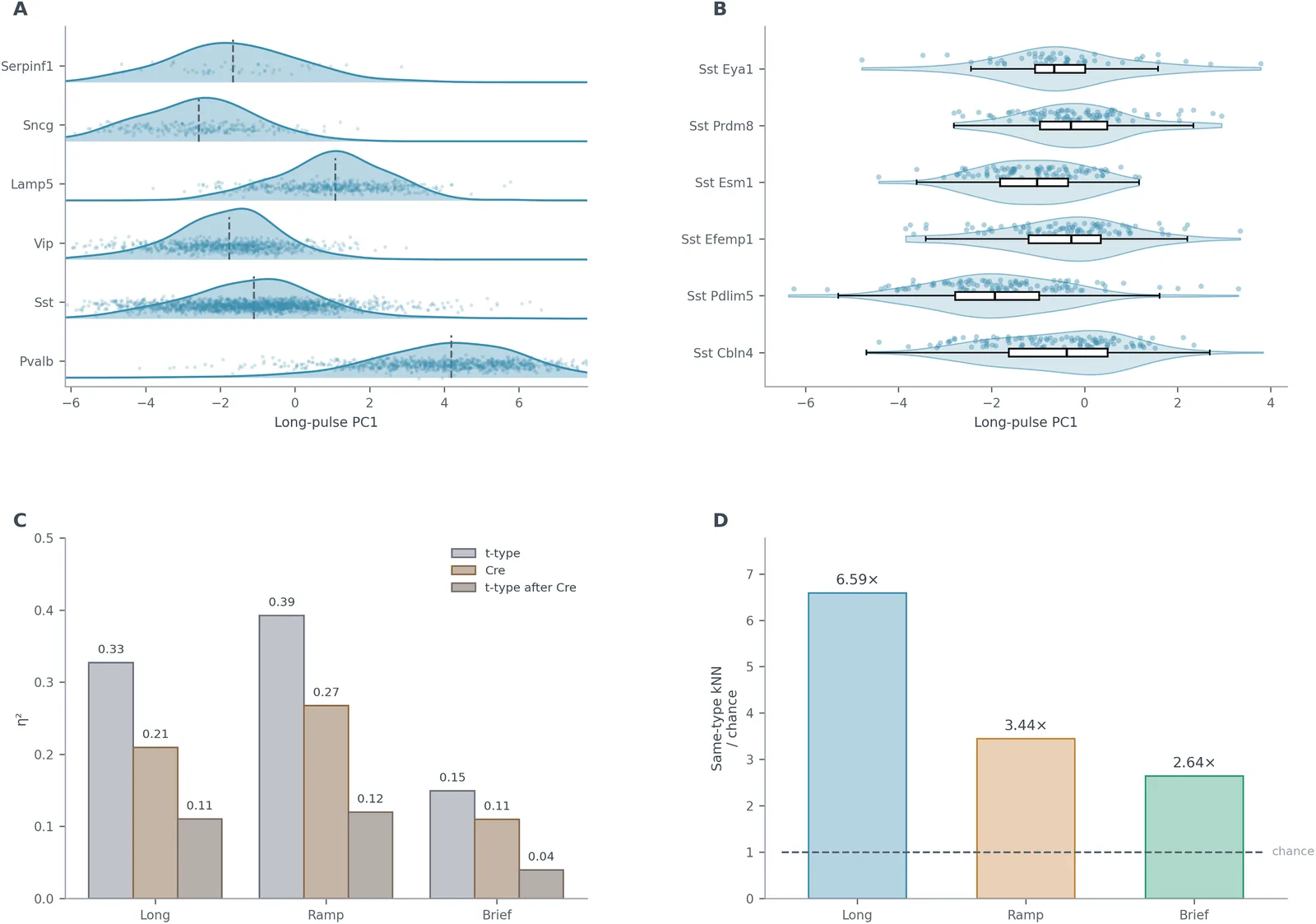
Transcriptomic type anchors phenotype beyond Cre. ***A***, Subclass occupancy along long-pulse PC1 (ridge densities with median dashed lines and jittered cells; subclasses with ≥8 cells). ***B***, Within the Sst-IRES-Cre driver, the six most frequent t-types still occupy distinct positions on long-pulse PC1 (horizontal raincloud: violin, box, jittered points). ***C***, Multivariate *η*^2^ of phenotype scores explained by t-type, Cre, and t-type after within-Cre residualization, for each regime. ***D***, Enrichment of same t-type among 15 nearest phenotypic neighbors relative to chance (dashed line = 1). Cell *n* with t-type: long-pulse 4,183; ramp 3,988; brief-pulse 4,266. Binary t-type–phenotype alignment (*A_t_*; *A^p^* denotes the corresponding statistic for a general label) and additional within-Cre analyses are reported in Extended Data Figure 2-1, not in this panel. See also Extended Data Figure 1-2.

10.1523/ENEURO.0275-26.2026.f2-1 Figure 2-1 **Anchoring controls. A**, Distance alignment: A_t_ (t-type), A^p^ (Cre), A^p^ (hierarchical subtype), and A_t_ (t-type after Cre). **B**, Within-Cre η² of t-type for long-pulse across the ten Cre lines with the most cells (labels include n). Download Figure 2-1, TIF file.

T-type accounted for more multivariate phenotypic variance than Cre identity in every response regime (*η*^2^ = 0.327 vs 0.209 long-pulse; 0.393 vs 0.267 ramp; 0.149 vs 0.110 brief-pulse; Fig. 2*C*), and residual t-type structure remained detectable after removing Cre-associated structure in all three regimes. Cells were also enriched for neighbors sharing their t-type relative to chance, with enrichment strongest for long-pulse (6.59-fold), followed by ramp (3.44-fold) and brief-pulse (2.64-fold; Fig. 2*D*).

Pairwise transcriptomic–phenotypic alignment was small in all three regimes (*A_t_* = 0.021^d^, 0.025^e^, and 0.021^f^ for long-pulse, ramp, and brief-pulse, respectively) but consistently exceeded shuffled controls, and this alignment remained above the shuffled-label null, though weaker and, for brief-pulse, very small (*A_t_* = 0.011^g^, 0.010^h^, 0.004^i^; Extended Data Fig. 2-1). Thus, transcriptomic type anchors cellular phenotype across response regimes without specifying the detailed position of individual cells: t-type—the more granular classification relative to Cre-driver identity (*η*^2^ = 0.327 vs 0.209 long-pulse; Fig. 2*C*)—remained the stronger correlate of phenotypic organization throughout, though weaker under brief-pulse stimulation.

### Type-level phenotypic relationships form a shared map with selective rewrites

We next shifted from cell–cell relationships to the organization of transcriptomic types. Analyses were restricted to 57 t-types represented by at least 10 cells in each response regime, yielding 1,596 shared type pairs. For each regime, we calculated distances between t-type centroids in phenotype space and compared the resulting type–type relational maps across regimes (Fig. 3*A*).

**Figure 3.**
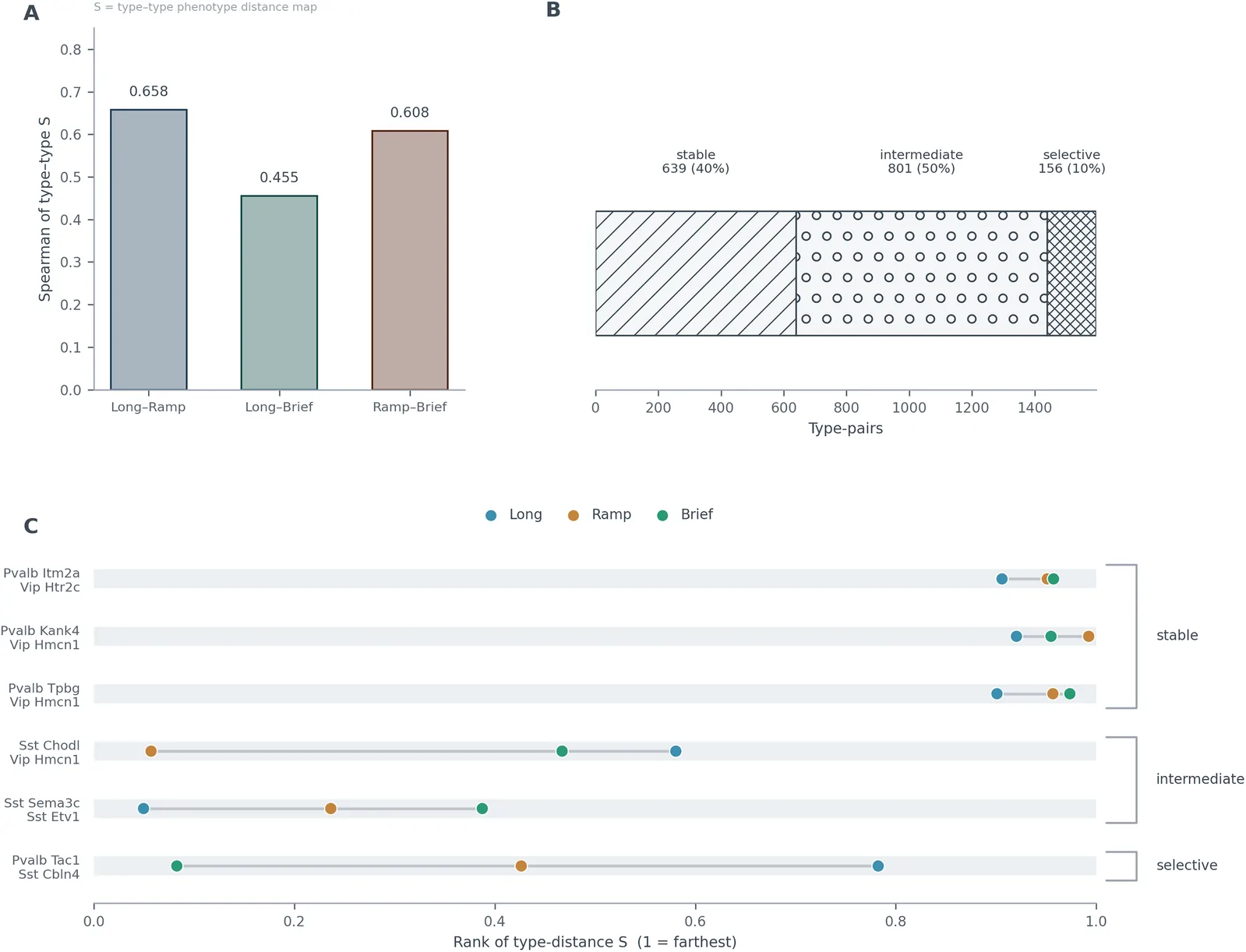
Shared type–type backbone with selective rewrites. ***A***, Spearman concordance of type–type phenotype distance maps *S_p_* across regimes for 57 t-types with ≥10 cells in every regime (*n* = 1,596 unordered pairs); empirical *p* = 0.000999 versus type-label shuffle for each protocol pair. Bars use the same muted pair hues as Figure 1. ***B***, Stacked counts of pair tags from rank-normalized *S* across the three regimes: stable (rank CV < 0.25; sparse slash), intermediate (open dots), selective (rank range ≥0.55; dense grid); counts and percentage of all pairs are shown (percentages rounded to the nearest integer). ***C***, Example type pairs (grouped by tag) showing rank of *S* in long-pulse, ramp, and brief-pulse (colored dots; 1, farthest); group labels at the right. See also Extended Data Figures 3-1–3-3.

10.1523/ENEURO.0275-26.2026.f3-1 Figure 3-1 **t-type counts.** Per t-type cell counts used for type–type analyses (eligibility and centroid construction). Download Figure 3-1, DOCX file.

10.1523/ENEURO.0275-26.2026.f3-2 Figure 3-2 **Type–type S maps and tag sensitivity. A–C**, Full 57 × 57 type–type distance maps S (Euclidean centroid distance in k_80_ phenotype space) for long-pulse, ramp, and brief-pulse; color scale = type–type D, with a separate scale per regime. **D**, Fraction of pairs tagged selective as the selective rank-range cutoff varies. **E**, Fraction tagged stable as the stable rank-CV cutoff varies. **F**, Spearman concordance of subclass-level S for the three protocol pairs. Download Figure 3-2, TIF file.

10.1523/ENEURO.0275-26.2026.f3-3 Figure 3-3 **Centroid robustness under equal-N resampling and split-half reliability. A**, Cross-regime S concordance from equal-N centroids (10 cells per type per regime; 200 resamples), shown as mean ± 95% interval, versus full-sample estimates, for each regime pair. **B**, Within-regime split-half S reliability (200 resamples) for each regime; the dashed line marks the mean full-sample cross-regime concordance (≈0.57) as a sampling-noise reference. Download Figure 3-3, TIF file.

Type-level phenotypic relationships were substantially concordant across all three regime pairs. Concordance was strongest between long-pulse and ramp responses (Spearman's *ρ* = 0.658^j^, *n* = 1,596 type pairs, empirical *p* = 0.000999), followed by ramp and brief-pulse responses (*ρ* = 0.608^k^, empirical *p* = 0.000999) and long-pulse and brief-pulse responses (*ρ* = 0.455^l^, empirical *p* = 0.000999; Fig. 3*A*). All three comparisons satisfied our prespecified criterion for a shared-map model (minimum *ρ* ≥ 0.40 and range <0.25). At the broader subclass level, phenotypic relationships were even more strongly conserved between long-pulse and ramp responses (*ρ* = 0.936, *n* = 6 subclasses) and remained moderately aligned for comparisons involving brief-pulse stimulation (ρ = 0.579 and 0.621; Extended Data Fig. 3-2*F*). Because unequal per-type sampling could in principle inflate this concordance, we additionally rebuilt centroids from equal-N draws (10 cells per type per regime; 200 resamples): cross-regime concordance decreased relative to the full-sample estimates for all three regime pairs (long-pulse–ramp: 0.658→mean 0.554, 95% interval 0.475–0.624; ramp–brief: 0.608→mean 0.455, 95% interval 0.353–0.539; long-pulse–brief: 0.455→mean 0.329, 95% interval 0.231–0.419), with the long-pulse–brief mean falling below the 0.40 threshold used for the shared-map criterion above, though its 95% interval still just overlaps that threshold (we do not apply the shared-map classification to these equal-N estimates). All three nonetheless remained well above shuffled-label nulls, indicating that type-level conservation is not accounted for solely by unequal sampling (Extended Data Fig. 3-3*A*). Within-regime split-half *S* concordance was substantially higher than the mean full-sample cross-regime value (long-pulse 0.861, ramp 0.851, brief-pulse 0.799; 200 resamples; Extended Data Fig. 3-3*B*), indicating that the shared-map estimates sit below the sampling-reliability ceiling rather than at it.

Shared organization did not imply invariance of every type relationship. We therefore rank-normalized type–type distances within each response regime and classified individual type pairs using prespecified criteria for cross-regime variability. Of the 1,596 type pairs, 639 (40.0%) were classified as stable, 801 (50.2%) were classified as intermediate, and 156 (9.8%) were classified as regime-selective (Fig. 3*B*). Representative trajectories illustrated both relationships that remained at similar relative distances under all three response regimes and a smaller subset whose relative positions shifted substantially (Fig. 3*C*). The proportions assigned to these categories were stable under modest variation of the classification thresholds (Extended Data Fig. 3-2*D*,*E*).

Together, these results support a shared type-level phenotypic backbone with selective regime-dependent rewrites superimposed on that common structure. Type-level conservation was substantially more apparent than preservation of local cell-level neighborhoods, indicating that biological organization can remain stable even when its detailed expression at the level of individual cells changes with the response regime.

### Transcriptomic structure preferentially tracks the shared phenotypic map

Having identified a shared type-level phenotypic backbone together with a smaller regime-selective component, we asked whether these two aspects of cellular organization were similarly reflected in transcriptomic structure. Using the primary transcriptomic representation, in which 500 genes were reduced to 10 principal components, we calculated transcriptomic distances *T* between t-type centroids and compared these distances with two complementary properties of the electrophysiological type map: cross-regime mean phenotypic separation, capturing the component of type-level separation expressed on average across regimes, and the cross-regime rank range of phenotypic-distance ranks, quantifying regime-selective reorganization.

Transcriptomic distance tracked cross-regime mean phenotypic separation much more strongly (Spearman's *ρ* = 0.507^m^, empirical *p* = 0.000999; Fig. 4*A*) than regime-selective rank range (*ρ* = 0.083^n^, *p* = 0.000999; Fig. 4*B*). This asymmetry was reproduced using two alternative transcriptomic feature sets: a curated 263-gene panel (*ρ* = 0.551^o^ vs 0.011^q^) and an independently defined set of highly variable genes (*ρ* = 0.520^p^ vs 0.105^r^; Extended Data Fig. 4-2*A*,*B*)—indicating that transcriptomic structure preferentially reflects the shared type-level backbone rather than the magnitude of regime-selective rewriting. Both the shared and regime-selective primary-panel correlations remained detectable under a higher-resolution 1,000-permutation null (rows m–n), underscoring that the asymmetry is best read from the effect sizes themselves (*ρ* = 0.507 vs 0.083) rather than from significance alone.

**Figure 4.**
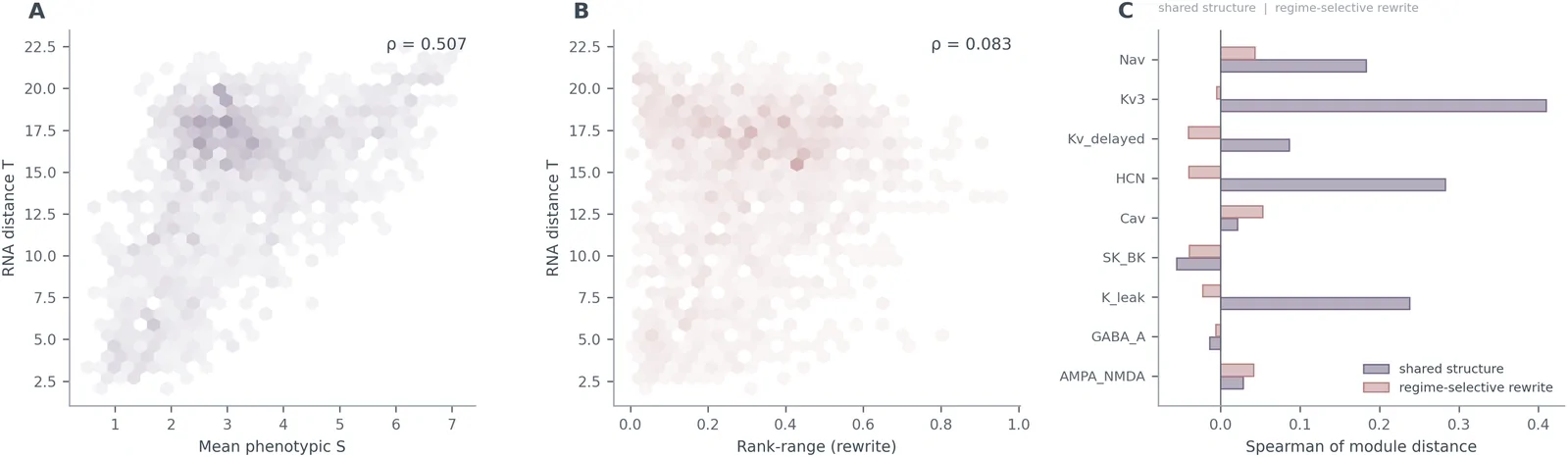
Transcriptomic geometry tracks shared structure, not rewrites. ***A***, Hexbin of RNA type–type distance *T* (primary 500-gene panel, 10 PCs) versus cross-regime mean phenotypic separation; Spearman *ρ* inset (*n* = 1,596 pairs; emp. *p* = 0.000999). ***B***, Same *T* versus rank range of rank-normalized *S* (regime-selective reorganization); *ρ* inset (emp. *p* = 0.000999). ***C***, Prespecified excitability modules: Spearman correlation of module type–type distance with cross-regime mean phenotypic separation (shared structure, purple) and with rank range (regime-selective reorganization, rose). See also Extended Data Figures 4-1–4-4.

10.1523/ENEURO.0275-26.2026.f4-1 Figure 4-1 **Module gene sets.** Gene membership for pre-specified excitability modules and marker-control modules used in Figure 4 and Extended Data Figure 4-2. Download Figure 4-1, DOCX file.

10.1523/ENEURO.0275-26.2026.f4-2 Figure 4-2 **Transcriptomic panels and marker controls. A**, Spearman ρ(T, mean S) for primary (500), curated (263), and HVG500 gene panels. **B**, Spearman ρ(T, rank-range) for the same panels. **C**, Subclass marker controls (Pvalb, Sst, Vip): Spearman correlation of marker-gene distance with mean S (shared structure) and rank-range (regime-selective rewrite). Download Figure 4-2, TIF file.

10.1523/ENEURO.0275-26.2026.f4-3 Figure 4-3 **WGCNA module–mobility association (unadjusted and t-type residual).** Spearman ρ of each of the ten WGCNA module eigengenes versus cell-level neighborhood mobility, before and after within-type centering. Unadjusted: *n* = 3,906 cells with matched RNA and transcriptomic type. Residual: *n* = 3,902 cells in types with ≥3 members (a subset of the 3,906). Empirical *p* from 1,000 label shuffles. The same contrasts are plotted in Extended Data Figure 4-4B. Download Figure 4-3, DOCX file.

10.1523/ENEURO.0275-26.2026.f4-4 Figure 4-4 **Cell-level mobility and module associations after t-type residualization. A**, Distribution of neighborhood mobility (1 − mean Spearman correlation of each cell's rank-distance profile across the three k_80_ regime pairs) for cells present in all three regimes (*n* = 4,049); dashed line marks the mean. **B**, Spearman ρ of module eigengenes versus mobility, unadjusted (filled; *n* = 3,906 cells with matched RNA and transcriptomic type) and after within-type centering of both the eigengene and mobility (open; *n* = 3,902 cells in types with ≥3 members, a subset of the 3,906). Ten unsigned WGCNA modules (Langfelder and Horvath, 2008; 2,000-gene panel, β = 3, scale-free R² = 0.891). Download Figure 4-4, TIF file.

Prespecified ion-channel and receptor gene sets reproduced this asymmetry. Kv3 expression showed the strongest association with mean phenotypic distance among the tested channel programs (*ρ* = 0.410), and a joint excitability-module representation correlated with mean phenotypic distance (*ρ* = 0.314^s^, empirical *p* = 0.020) but not with rank range (*ρ* = −0.023^t^, *p* = 0.294; Fig. 4*C*). No tested excitability program, individual or joint, showed more than a negligible association with rank range (largest individual-module |*ρ*| = 0.053, Cav); subclass marker controls (Pvalb, Sst, Vip) are shown for comparison in Extended Data Figure 4-2*C*.

These module-level associations were not interpreted as causal mechanisms: a Pvalb marker control showed an even stronger correspondence with mean phenotypic distance (*ρ* = 0.543) than any individual excitability module, consistent with established identity axes contributing to both transcriptomic and electrophysiological organization.

As an Extended Data control, we asked whether within-type transcriptomic heterogeneity was associated with regime-dependent phenotypic reorganization beyond transcriptomic type identity. We scored each cell present in all three regimes by how much its relative *k*_80_ neighborhood changed across regimes (mobility = 1 − mean Spearman correlation of rank-distance profiles across the three regime pairs; *n* = 4,049; mean mobility = 0.589, SD = 0.110^x^). WGCNA modules built on the prespecified 2,000-gene panel showed modest associations with mobility before accounting for transcriptomic type (largest |*ρ*| = 0.199; Extended Data Fig. 4-3), but after within-type centering of mobility and module eigengenes, residual associations fell to |*ρ*| ≤ 0.039 for all modules, and no module retained both |*ρ*| ≥ 0.10 and empirical *p* < 0.05^y^ (*n* = 3,902 cells in types with ≥3 members; Extended Data Fig. 4-4). We therefore found little evidence that within-type transcriptomic heterogeneity in this panel was associated with regime-dependent neighborhood mobility beyond t-type identity; the principal molecular correspondence remained at the level of transcriptomic type.

Together, these results reveal an asymmetry in the molecular organization of multi-regime cellular phenotype: transcriptomic structure and predefined excitability-related programs preferentially recover the shared type-level phenotypic backbone, whereas the molecular dimensions examined here provide little explanatory leverage over regime-selective rewrites.

Exact sample sizes, effect sizes, and *p* values for every contrast reported with a specific empirical *p* value are provided in Table 2 (rows a–y). Letters match superscripts in Results. Empirical *p* = 0.0099 is the resolution of 100 shuffles when no null exceeds the observed statistic; *p* = 0.020 likewise for 50 shuffles, and *p* = 0.000999 for 1,000 shuffles (rows j–n). At this resolution a small effect (e.g., row n, *ρ* = 0.083) can reach the same floor *p* value as a large one (row m, *ρ* = 0.507); effect size, not significance, distinguishes them. Split-half and *k*-sweep rows (u–w) use 100 index shuffles; WGCNA (row y) uses 1,000 label shuffles. A complete inventory of statistics, including null statistics where retained and descriptive effect sizes without permutation-based *p*-values, is provided in Extended Data Table 2-1.

## Discussion

Our results reveal a separation between the stability of cellular electrophysiological phenotype and the stability of the biological organization in which that phenotype is embedded. Across long-pulse, ramp, and brief-pulse response regimes, relationships among individual cells were only partially conserved: global pairwise distances retained measurable correspondence, but local cellular neighborhoods were much less stable, a pattern that persisted across a broad range of neighborhood scales and that could not be fully accounted for by the variability observed between independent measurements within a single response regime. In contrast, transcriptomic type remained associated with phenotype in every regime, and relationships among transcriptomic types formed a substantially more conserved relational map. Molecular similarity, in turn, preferentially corresponded to this shared type-level organization rather than to the subset of relationships that changed across regimes. Together, these findings suggest that variation in how cellular phenotype is expressed across stimulation conditions can coexist with a more stable underlying biological organization.

### Flexible cellular phenotype remains biologically anchored

Electrophysiological phenotype should not be treated as a single fixed arrangement of cells. The three response regimes examined here emphasized different aspects of intrinsic physiology and produced phenotype spaces of markedly different dimensionality, and the same cells did not preserve identical relationships across these spaces: global pairwise-distance correlations remained moderate across regimes, and local nearest-neighbor overlap was minimal. Because principal components were fit independently within each regime, we do not treat the leading phenotypic axis as directly comparable across regimes; the features loading most strongly onto it differ by regime (Extended Data Fig. 1-2*D*–*F*). All three cross-regime distance correlations nonetheless exceeded shuffled expectations, indicating that the cross-regime relationships retain structured correspondence rather than reflecting arbitrary organization: different current-clamp regimes are best viewed as complementary probes of cellular phenotype rather than interchangeable measurements of a single electrophysiological coordinate system.

This local reorganization was not specific to a single neighborhood size. Repeating the analysis across neighborhood sizes from *k* = 15 to *k* = 600, chance-adjusted overlap remained limited across all scales tested and increased only gradually as progressively broader neighborhoods were considered (Extended Data Fig. 1-2*B*,*C*). This incomplete recovery indicates that cross-regime reorganization extends beyond the immediate local neighborhood rather than reflecting an idiosyncrasy of the smallest scale. At the same time, the persistence of moderate global pairwise-distance correlations (above) indicates that this reorganization does not imply an absence of shared phenotypic structure.

This reorganization also cannot be attributed solely to trial-to-trial measurement variability. Within-regime neighborhoods reconstructed from independent halves of long-pulse sweeps were substantially more reproducible than neighborhoods compared across response regimes, although within-regime reproducibility remained incomplete (Extended Data Fig. 1-3). Cross-regime reorganization therefore exceeds the variability observed between repeated measurements within a single regime while remaining superimposed on appreciable trial-level variability. Because sufficiently repeated sweeps were available only for the long-pulse protocol, comparable within-regime reliability could not be assessed for ramp or brief-pulse responses.

The persistence of transcriptomic organization across all three response regimes provides a complementary perspective on this flexibility. Previous Patch-seq studies established extensive correspondence between transcriptomic identity and electrophysiological or morphoelectric phenotype (Gouwens et al., 2019, 2020; Lee et al., 2021; Scala et al., 2021), while other work related gene-expression variation to intrinsic electrophysiological diversity (Tripathy et al., 2017; Bomkamp et al., 2019). Our results do not redefine this correspondence; rather, they show it remains detectable when electrophysiological phenotype is resolved separately under distinct response regimes. T-type explained more phenotypic variance than Cre identity in every regime, and cells were consistently enriched for neighbors sharing their t-type, yet pairwise transcriptomic–phenotypic alignment was weak: transcriptomic identity does not specify the detailed position of every cell in phenotype space. Instead, transcriptomic identity biases the regions of phenotype space that cells occupy and the probability that phenotypically neighboring cells share biological identity.

This distinction between anchoring and specification may help reconcile two observations that can otherwise appear contradictory: electrophysiological variability within transcriptomically defined types and reproducible multimodal differences among types. Transcriptomic identity can constrain phenotype without determining a unique electrophysiological state, consistent with broader evidence that similar neuronal output can arise from variable combinations of intrinsic conductances (Marder and Goaillard, 2006; Schulz et al., 2006; Taylor et al., 2009)—response regime modulates the expression of cellular phenotype within a biologically structured landscape rather than erasing the landscape itself.

Within-type transcriptomic heterogeneity showed little association with regime-dependent phenotypic mobility beyond t-type identity: gene coexpression modules showed modest associations with mobility before accounting for t-type, but these were strongly attenuated once t-type-associated variation was removed (Extended Data Fig. 4-4). This result is limited to the 2,000-gene panel examined here and does not exclude contributions from other within-type molecular variation or regulatory levels not captured by transcript abundance.

Several biological layers not captured by transcript abundance could mediate this distinction between molecular identity and phenotypic expression. Transcript levels do not directly specify the abundance or subcellular localization of functional ion channels and signaling molecules; posttranscriptional and posttranslational regulation may further shape channel properties, trafficking, and membrane insertion; and a cell's prior activity and physiological state may influence which conductances are engaged under a given stimulation regime. Cellular morphology provides an additional layer of constraint: dendritic and axonal architecture shape electrotonic structure and the spatial distribution of membrane conductances and could contribute both to the relatively stable organization associated with cell type and to regime-dependent electrophysiological expression through its interaction with the subcellular localization of ion channels and other membrane components. Morphology was not explicitly incorporated into the present framework, which was designed to isolate relationships between electrophysiological response regimes and transcriptomic identity. Integrating morphology, transcriptomic state, and regime-specific electrophysiology within a common relational framework will be important for determining which layers of cellular organization underlie stable versus flexible components of phenotype.

### Type-level organization is more stable than its cellular expression

The strongest evidence for this separation emerged when the analysis moved from individual cells to relationships among transcriptomic types. Although local cell neighborhoods were substantially reorganized across response regimes, type-centroid relationships remained substantially concordant, and the broader subclass organization was more conserved still. Individual cells can move relative to one another across response regimes while population-level relationships among biological types remain recoverable: type identity provides a relational scaffold rather than a fixed cellular coordinate, and the electrophysiological expression of a type can vary with the stimulus used to interrogate the cell even while the relative organization among types remains comparatively stable. This type-level conservation was reduced when centroids were estimated from equal numbers of cells per type, particularly for the weakest long-pulse–brief comparison, but remained above shuffled expectations (Extended Data Fig. 3-3*A*). Sampling therefore contributes to the magnitude of type-level concordance, but does not fully account for the broader separation between type-level and cell-level organization.

A minority of type pairs remained regime-selective, so the shared type-level map was not completely invariant. This pattern is best described as selective reorganization superimposed on a broadly shared relational backbone—neither an immutable type hierarchy nor a set of unrelated, regime-specific organizations.

We used geometric quantities—distances, neighborhoods, centroids, and relational maps—as a common descriptive language for comparing organization across response regimes and biological levels, rather than as mechanistic objects. The relevant distinction was therefore not the interpretation of individual principal components, but the scale at which relational organization persists.

### Molecular structure preferentially reflects the shared component

Separating shared from regime-selective type-level structure allowed us to ask a more specific molecular question than whether transcriptomic and electrophysiological phenotypes are correlated in general. Transcriptomic distance tracked cross-regime mean phenotypic separation much more strongly than regime-selective rank range, and this asymmetry was reproduced using alternative transcriptomic feature sets. Transcriptomic organization therefore corresponded much more strongly to the component of electrophysiological organization that was repeatedly recovered across response regimes than to the component that changed among them.

Prespecified excitability-related molecular programs showed a similar asymmetry: Kv3 expression, which shapes fast-repolarizing firing properties in cortical interneurons (Rudy and McBain, 2001), was associated with the shared phenotypic map but not with regime-selective rank changes, and no tested excitability program showed more than a negligible association with rank range. These associations were not interpreted as causal mechanisms: as noted above, the Pvalb marker control exceeded the correspondence of any individual excitability module, indicating that these relationships partly reflect broader molecular distinctions among neuronal types rather than a specific channel mechanism. More generally, intrinsic electrophysiological properties typically arise from combinations of interacting conductances rather than one-to-one mappings between individual genes and physiological features (Marder and Goaillard, 2006; Taylor et al., 2009), consistent with the absence of a single dominant channel correlate of regime-selective rewriting here.

The absence of a comparable molecular account for regime-selective rewriting is itself informative. Selective type-pair changes were clearly detectable electrophysiologically, but neither transcriptomic distance nor the predefined molecular programs examined here showed substantial correspondence with which relationships were most strongly rewritten. Molecular correspondence is therefore not uniform across all components of electrophysiological phenotype: the shared structure is considerably more molecularly legible than its regime-dependent modification.

### Limitations

The transcriptomic and electrophysiological representations compared here differ substantially in dimensionality and construction: transcriptomic measurements span hundreds to thousands of genes, whereas the electrophysiological representations used in this study comprise tens of engineered response features per regime. This asymmetry could in principle influence which aspects of cellular identity each modality is able to resolve. However, the same transcriptomic representation, held fixed throughout, was substantially more strongly related to the shared than to the regime-selective component of electrophysiological organization (*ρ* = 0.507 vs *ρ* = 0.083), and this asymmetry persisted across alternative gene panels and electrophysiological dimensionality choices. This pattern should not be interpreted as evidence that transcriptomic identity is intrinsically more complete or information-rich than electrophysiological phenotype.

This study is a secondary analysis of an observational Patch-seq dataset from mouse visual cortical GABAergic neurons, and the molecular associations reported here are therefore correlational: they do not establish that transcriptomic differences, Kv3 expression, or any other molecular program causally generate the corresponding electrophysiological organization. The original dataset also reflects nonrandom sampling through Cre-driver lines, and its composition should not be interpreted as an unbiased census of cortical interneuron diversity.

The three response regimes differ in both stimulus structure and the electrophysiological features that can be extracted from them, so their phenotype spaces differ in dimensionality and feature content. This is intrinsic to the question of protocol-resolved phenotype but means that cross-regime differences cannot be assigned to a single underlying physiological process; the fixed-component sensitivity analysis supports the robustness of the broad cross-regime ordering but does not make the three feature spaces equivalent.

Because type-level analyses average over within-type heterogeneity, centroid averaging could inflate apparent stability through noise reduction rather than a genuine biological difference between cell-level and type-level conservation. Two controls helped assess the contribution of sampling and measurement variability to this difference. Equal-N resampling (10 cells per type per regime) preserved the overall type-level pattern, although the weakest long-pulse–brief comparison was sensitive to reduced sampling and fell below the predefined shared-map threshold (Extended Data Fig. 3-3*A*). A complementary cell-level split-half analysis of long-square sweeps showed substantially greater within-regime than cross-regime neighborhood overlap, though trial-to-trial variability remained appreciable (Extended Data Fig. 1-3). Together, these analyses indicate that sampling and measurement variability contribute to, but do not fully explain, the greater conservation observed at the type level. The full-sample estimates should nevertheless be interpreted as a modest upper bound on type-level stability rather than evidence that individual cells possess invariant type-specific phenotypes.

Finally, the molecular analysis was restricted to predefined transcriptomic representations and biologically motivated gene programs, leaving the molecular basis of regime-selective rewriting unresolved.

Together, these results indicate that cellular electrophysiological phenotype is context-dependent but not biologically unstructured. Relationships among individual cells reorganize substantially across response regimes, yet type-level organization persists far more strongly, and transcriptomic similarity preferentially reflects this shared component rather than its regime-selective modification. Characterizing neuronal phenotype across multiple response regimes may therefore reveal not only how cells differ, but which components of those differences constitute stable biological organization and which emerge only under particular conditions.

## Synthesis

Reviewing Editor: Rishikesh Narayanan, Indian Institute of Science Decisions are customarily a result of the Reviewing Editor and the peer reviewers coming together and discussing their recommendations until a consensus is reached. When revisions are invited, a fact-based synthesis statement explaining their decision and outlining what is needed to prepare a revision will be listed below. The following reviewer(s) agreed to reveal their identity: Nicolas Gervasi.

In this study, the author investigates if specific aspects of cellular electrophysiological organization persist across distinct response regimes and how this persistence relates to transcriptomic identity. The author uses the Allen Institute dataset from Gouwens et al. (2020) showing that cell-level phenotypic relationships were only partially conserved across regimes, whereby global pairwise structure remained detectable but dominant phenotypic axes and local neighborhoods were substantially reorganized. Together, the author reports that individual cellular relationships were flexible across probes, whereas type-level biological organization was comparatively stable and was relatively strongly reflected in transcriptomic structure.

The reviewers were appreciative of the specific questions posed, the framework for addressing the questions, and the conclusions drawn. The reviewers considered the question to be important from both methodological and technical standpoints, probing the biological basis of electrophysiological variation across response regimes as well as assessing dimensions to explore correlations between transcriptomic structure and electrophysiological properties. They were particularly enthusiastic about the distinction that the author offers between conservation of the global/type-level organization and flexibility/plasticity of individual-cell relationships.

Despite the overall positive reviews, the reviewers expressed the need for additional analyses and provided important comments to improve methodological clarity, interpretations, and presentation of the manuscript. These reviewer comments are synthesized below and the individual reviews are copied at the end of the synthesis.

1. Please repeat the nearest neighbor analyses with different (higher) values for the range of neighborhood sizes.

2. Please perform split-half analyses at the level of individual cells.

3. Please account for single cell transcriptomic heterogeneity to identify potentially coordinated transcriptional programs associated with phenotypic flexibility. Discuss the possibility that a neuron's transcriptome constrains its phenotypic expression without uniquely specifying its phenotype, bringing in other variations including differential subcellular localization of transmembrane and cytosolic components.

4. Please clarify interpretation of the first principal component (PC1) across different electrophysiological response regimes. Please report loadings of the main features contributing to PC1 for all three regimes.

5. Please clarify why cellular morphology was not considered in the analyses here. Cellular morphology is an extremely important determinant of electrophysiological characteristics, by itself and in terms of forming the substrate for differential subcellular localization of individual components (mentioned in #3 above). Please provide clear rationale.

6. Please discuss the impact of the large difference in dimensionality between transcriptomic and electrophysiological measurements on the conclusions drawn from the analyses. Could the much higher-dimensional transcriptomic representation capture a considerably richer description of cellular identity than the electrophysiological feature?

7. Please consider having a more consistent color code across figures. Please consider reorganizing figures to make the points more clearer.

All specifics are provided below within the individual reviews. Please provide point-by-point responses to each review point from the individual reviews, which will allow the reviewers to re-review the manuscript and adjudge the suitability of the manuscript. Please also revise the extended data figures and analyses code files appropriately in the revised version.

**REVIEWERS' COMMENTS**

**COMMENTS BY REVIEWER #1**

**Advances the Field (Required)**

The authors address a fascinating and increasingly important questions at mechanistic and technical levels; Mechanistically, regarding the biological basis of electrophysiological variation across response regimes; and technically, concerning the value / limitations /appropriate scope of transcriptomic-electrophysiological correlations. As a whole, the study provides a thoughtful framework for addressing these questions. The distinction they draw between the stability at the level of transcriptomic types and flexibility at the level of single-cell electrophysiological phenotype is interesting and raises questions about the scale at which neuronal identity is stable versus context dependent.

**Comments to the Authors (Required)**

The authors address a fascinating and increasingly important question at mechanistic and technical levels; Mechanistically, regarding the biological basis of electrophysiological variation across response regimes; and technically, concerning the value / limitations /appropriate scope of transcriptomic-electrophysiological correlations. As a whole, the study provides a thoughtful framework for addressing these questions. Additionally, the text of the manuscript is well-written and should be accessible to both experts and non-experts. However, additional analyses would help strengthen the conclusions regarding phenotypic stability and reorganization (see Recommendations 1 and 2).

The data also raises an intriguing possibility that a neuron's transcriptome constrains its phenotypic expression without uniquely specifying its phenotype. Even among cells with the same transcriptomic identity, electrophysiological phenotype could depend on other sources of variation like ion-channel and signaling-molecule localization to different subcellular compartments, post-translational modifications of channels and receptors, and other processes shaped by the prior history of the cell. The authors should discuss these possibilities more explicitly. However, before such conclusions regarding the limitations of the transcriptomic identity can be drawn, the authors should test whether molecular heterogeneity within transcriptomic types is associated with regime-dependent phenotypic flexibility (see Recommendation 3).

Recommendations to the authors:

1. In the nearest-neighbor analysis, the authors use k=15, which makes it difficult to determine the scale at which cross-regime organization is preserved or lost. The authors should repeat this analysis across a range of neighborhood sizes (e.g., k=15, 30, 60, ..., 600). This analysis can help to reveal whether the low overlap at k=15 reflects highly local rearrangement, or whether reorganization persists across broader regions of phenotypic space. Because expected Jaccard overlap will increase with k even under random organization, observed values should be compared with the priper chance-adjusted values at each value of k. This would provide a much clearer picture of the spatial scale at which phenotypic organization becomes stable across response regimes.

2. The authors use split-half analyses to address whether greater stability at the type level could be just from noise reduction through averaging, but this analysis is performed at the centroid level. The argument would be stronger at the level of individual cells. The authors could use stimulus-matched sweeps in the dataset to ask how reliably independent trials place a given cell within its local phenotypic neighborhood and then compare this within-regime variability with the reorganization observed between regimes. If within-regime neighborhoods are highly reproducible compared to cross-regime neighborhoods it would provide stronger evidence that the effect reflects stimulus-dependent changes in phenotypic expression rather than trial-to-trial variability. On the other hand substantial within-regime reorganization would recommend a more cautious interpretation of the cross-regime effect.

3. The current molecular analysis is performed at the level of t-type centroids and gene panels which may obscure single-cell transcriptomic heterogeneity associated with regime-dependent reorganization. I suggest defining a continuous "mobility score" for each cell based on how strongly its relative position in phenotype space changes across regimes and using this score as a "trait" in weighted gene co-expression network analysis (WGCNA). Correlating module eigengenes with mobility could identify coordinated transcriptional programs associated with phenotypic flexibility. These associations should be tested with t-type as a covariate to distinguish molecular correlates of regime-dependent mobility from transcriptomic identity.

**COMMENTS BY REVIEWER #2**

**Advances the Field (Required)**

This study provides interesting advance by showing that the organization of neuronal electrophysiological phenotypes is only partially stable at different levels of analysis. The comparison between individual cell relationships, transcriptomic genotype and different electrophysiological response regimes gives a useful view on how a relatively stable biological organization can coexist with changes in the expression of cellular phenotype.

**Comments to the Authors (Required)**

This study entitled : Flexible cellular phenotypes retain a shared type-level organization across response regimes makes use of the Allen Institute dataset from Gouwens et al. (2020), Integrated morphoelectric and transcriptomic classification of cortical GABAergic cells, Cell 183:935-953, to address an important question on how cellular electrophysiological organization persist across distinct response regimes and how this persistence relates to transcriptomic identity? The question is relevant, and examine the organization of cellular phenotypes at multiple scales, from individual-cell electrophysiological relationships to transcriptomic type-level organization. The main conclusions show that transcriptomic t-types provide a relatively stable backbone of electrophysiological organization. And the detailed electrophysiological phenotype remains substantially plastic across response regimes. The distinction between conservation of the global/type-level organization and plasticity of individual-cell relationships is a valuable aspect of the study.

The analyses are extensive and well conducted. I nevertheless have several comments that can improve the methodological clarity, interpretation, and presentation of the manuscript.

1. Interpretation of PC1 across electrophysiological response regimes

Some conclusions and figures (like Figure 1B, C, 2A, 2B) relies on comparisons of PC1 across the three response regimes. These PCA are performed independently for each response regime, using regime-specific electrophysiological features. PC1 in one regime is not necessarily equivalent to PC1 in another regime and electrophysiological properties contributing could differ between them. The authors need to provide a clearer characterization of the PC1 obtained in each regime. Please report the loadings of the main features contributing to PC1 for all three regimes.

2. The role of cellular morphology is not considered

The dataset used in this study contains not only electrophysiological and transcriptomic information, but also detailed morphological characterization of the cells. The manuscript didn't use this component of the dataset from the Allen Brain Institute. I do not ask the authors to incorporate morphology into the main analyses. But i believe that morphology should at least be discussed as a potentially important factor underlying the observed electrophysiological variation across response regimes. Morphological properties, including dendritic and axonal architecture, can strongly influence the electrophysiological features measured from a neuron. Morphology itself may reflect both relatively stable cellular identity and context-dependent influences.

3. Clarification of the multivariate η² calculation

The manuscript uses "multivariate η²" to quantify the amount of phenotypic variance associated with t-type and Cre identity. The precise calculation of this statistic is not sufficiently described in the Methods.

4. Difference in dimensionality between transcriptomic and electrophysiological measurements

A point that would benefit from discussion concerns the substantial difference in dimensionality between the two biological modalities considered in the study. The electrophysiological phenotype is described using several tens of quantitative parameters, whereas transcriptomic identity is derived from the expression of several hundreds to thousands of genes. Could the much higher-dimensional transcriptomic representation capture a considerably richer description of cellular identity than the electrophysiological feature? And could contribute to the observed asymmetry between transcriptomic and electrophysiological phenotype.

5. Figure design and consistency of color coding

The figures would benefit from a more coherent visual design. I recommend assigning a specific color to each electrophysiological response regime and maintaining the same color coding throughout the entire manuscript. As an example, the three regimes are represented by different colors in different figures (Figures 1D, 2D, and 3A). The categories "stable," "intermediate," and "selective" in Figures 3B and 3C should also have a consistent color scheme between the two panels.

6. Figure 1 should be substantially revised

I find the current Figure 1 insufficiently aligned with the main message of the Results section. In particular, only panel 1D appears to be cited in the Results paragraph named : Cellular phenotypes are partially conserved and locally reorganized across response regimes, whereas the manuscript point to Extended Data Figures 1-1 and 1-2. The current Figure 1 does not appear to communicate the central result of this paragraph. Please change this figure to more directly represent the two main aspects of cross-regime phenotype conservation described in the manuscript and put reference to this figure in the main text. One possible organization could be: (A) representative electrophysiological responses illustrating the three response regimes, (B) global phenotypic organization, showing pairwise distance-distance representations between shared cells for each regime and bar graph of Spearman correlations and (C) local phenotypic organization, showing the distribution of Jaccard data of the 15 nearest neighbors across the three regime pairs. The current PC1 density (Figure 1B) and PC1 quintile-transfer panels (Figure 1C) rely on independently computed PCA axes making their biological interpretation difficult (see my comment 1). These representation could be remove without losing any information for the reader.
