# Supplementary material for: Flexible Cellular Phenotypes Retain a Shared Type-Level Organization across Response Regimes
Source: eNeuro. 2026 Nov 2;13(11):ENEURO.0275-26.2026. doi: 10.1523/ENEURO.0275-26.2026 (PMC13737997; doi:10.1523/ENEURO.0275-26.2026)
Supplement: Supplementary file 2 [file eneuro-13-ENEURO.0275-26.2026-s010.docx]

**Extended Data Figure 3-1. t-type counts.**

| t-type | n Long | n Ramp | n Brief |
| --- | --- | --- | --- |
| Lamp5 Fam19a1 Tmem182 | 22 | 22 | 22 |
| Lamp5 Krt73 | 12 | 12 | 12 |
| Lamp5 Lhx6 | 19 | 19 | 19 |
| Lamp5 Lsp1 | 186 | 193 | 193 |
| Lamp5 Ntn1 Npy2r | 72 | 71 | 73 |
| Lamp5 Plch2 Dock5 | 150 | 148 | 150 |
| Pvalb Akr1c18 Ntf3 | 64 | 57 | 64 |
| Pvalb Calb1 Sst | 38 | 28 | 38 |
| Pvalb Gabrg1 | 46 | 22 | 46 |
| Pvalb Gpr149 Islr | 24 | 22 | 24 |
| Pvalb Reln Itm2a | 236 | 216 | 240 |
| Pvalb Reln Tac1 | 45 | 39 | 45 |
| Pvalb Sema3e Kank4 | 175 | 153 | 176 |
| Pvalb Th Sst | 54 | 46 | 55 |
| Pvalb Tpbg | 173 | 161 | 176 |
| Pvalb Vipr2 | 22 | 24 | 24 |
| Serpinf1 Aqp5 Vip | 34 | 34 | 34 |
| Serpinf1 Clrn1 | 13 | 12 | 13 |
| Sncg Gpr50 | 49 | 49 | 49 |
| Sncg Slc17a8 | 12 | 12 | 12 |
| Sncg Vip Itih5 | 134 | 134 | 134 |
| Sncg Vip Nptx2 | 39 | 39 | 39 |
| Sst Calb2 Necab1 | 54 | 53 | 55 |
| Sst Calb2 Pdlim5 | 257 | 263 | 266 |
| Sst Chodl | 131 | 124 | 132 |
| Sst Chrna2 Glra3 | 19 | 18 | 19 |
| Sst Chrna2 Ptgdr | 10 | 10 | 10 |
| Sst Crh 4930553C11Rik | 42 | 37 | 42 |
| Sst Crhr2 Efemp1 | 116 | 108 | 116 |
| Sst Esm1 | 145 | 142 | 146 |
| Sst Hpse Cbln4 | 246 | 244 | 252 |
| Sst Hpse Sema3c | 56 | 56 | 58 |
| Sst Mme Fam114a1 | 68 | 59 | 68 |
| Sst Myh8 Etv1 | 60 | 60 | 61 |
| Sst Myh8 Fibin | 18 | 17 | 18 |
| Sst Nr2f2 Necab1 | 37 | 37 | 37 |
| Sst Nts | 47 | 32 | 47 |
| Sst Rxfp1 Eya1 | 96 | 89 | 96 |
| Sst Rxfp1 Prdm8 | 139 | 130 | 143 |
| Sst Tac1 Htr1d | 62 | 62 | 63 |
| Sst Tac1 Tacr3 | 53 | 50 | 54 |
| Sst Tac2 Myh4 | 29 | 29 | 30 |
| Sst Tac2 Tacstd2 | 111 | 109 | 111 |
| Vip Arhgap36 Hmcn1 | 36 | 33 | 36 |
| Vip Chat Htr1f | 61 | 63 | 63 |
| Vip Col15a1 Pde1a | 53 | 53 | 54 |
| Vip Crispld2 Htr2c | 47 | 47 | 48 |
| Vip Crispld2 Kcne4 | 102 | 85 | 107 |
| Vip Gpc3 Slc18a3 | 18 | 18 | 20 |
| Vip Igfbp4 Mab21l1 | 16 | 20 | 20 |
| Vip Lect1 Oxtr | 28 | 25 | 30 |
| Vip Lmo1 Fam159b | 39 | 35 | 39 |
| Vip Lmo1 Myl1 | 87 | 80 | 91 |
| Vip Ptprt Pkp2 | 123 | 125 | 127 |
| Vip Pygm C1ql1 | 94 | 95 | 99 |
| Vip Rspo1 Itga4 | 10 | 11 | 11 |
| Vip Rspo4 Rxfp1 Chat | 39 | 40 | 43 |

Per t-type cell counts used for type–type analyses. Related to Figure 3.
