# Supplementary material for: Flexible Cellular Phenotypes Retain a Shared Type-Level Organization across Response Regimes
Source: eNeuro. 2026 Nov 2;13(11):ENEURO.0275-26.2026. doi: 10.1523/ENEURO.0275-26.2026 (PMC13737997; doi:10.1523/ENEURO.0275-26.2026)
Supplement: Supplementary file 3 [file eneuro-13-ENEURO.0275-26.2026-s011.docx]

**Extended Data Figure 4-1. Module gene sets.**

| Module | Role | n genes | Genes |
| --- | --- | --- | --- |
| Nav | Excitability | 9 | Scn1a, Scn2a1, Scn3a, Scn8a, Scn9a, Scn1b, Scn2b, Scn3b, Scn4b |
| Kv3 | Excitability | 4 | Kcnc1, Kcnc2, Kcnc3, Kcnc4 |
| Kv_delayed | Excitability | 7 | Kcna1, Kcna2, Kcnb1, Kcnb2, Kcnq2, Kcnq3, Kcnq5 |
| HCN | Excitability | 4 | Hcn1, Hcn2, Hcn3, Hcn4 |
| Cav | Excitability | 8 | Cacna1a, Cacna1b, Cacna1c, Cacna1d, Cacna1e, Cacna1g, Cacna1h, Cacna1i |
| SK_BK | Excitability | 7 | Kcnn1, Kcnn2, Kcnn3, Kcnma1, Kcnmb1, Kcnmb2, Kcnmb4 |
| K_leak | Excitability | 7 | Kcnk1, Kcnk2, Kcnk3, Kcnk9, Kcnj2, Kcnj4, Kcnj12 |
| GABA_A | Excitability | 11 | Gabra1, Gabra2, Gabra3, Gabra4, Gabra5, Gabrb1, Gabrb2, Gabrb3, Gabrg1, Gabrg2, Gabrg3 |
| AMPA_NMDA | Excitability | 9 | Gria1, Gria2, Gria3, Gria4, Grin1, Grin2a, Grin2b, Grin2c, Grin2d |
| Pvalb | Marker control | 2 | Pvalb, Tac1 |
| Sst | Marker control | 4 | Sst, Crh, Cort, Chodl |
| Vip | Marker control | 3 | Vip, Cck, Calb2 |

Gene membership for pre-specified excitability modules and marker-control modules used in Figure 4 and Extended Data Figure 4-2.
