# Supplementary material for: Flexible Cellular Phenotypes Retain a Shared Type-Level Organization across Response Regimes
Source: eNeuro. 2026 Nov 2;13(11):ENEURO.0275-26.2026. doi: 10.1523/ENEURO.0275-26.2026 (PMC13737997; doi:10.1523/ENEURO.0275-26.2026)
Supplement: Supplementary file 4 [file eneuro-13-ENEURO.0275-26.2026-s012.docx]

**Extended Data Figure 4-3. WGCNA module–mobility association (unadjusted and t-type residual).**

| Module | n genes | Hub | Spearman ρ unadjusted | Empirical p unadjusted | Spearman ρ t-type residual | Empirical p t-type residual |
| --- | --- | --- | --- | --- | --- | --- |
| red | 67 | Erbb4 | -0.199 | 0.001 | -0.039 | 0.021 |
| magenta | 52 | Grin3a | 0.192 | 0.001 | -0.021 | 0.204 |
| blue | 181 | Sox6 | 0.089 | 0.001 | 0.013 | 0.430 |
| black | 65 | Dscam | 0.089 | 0.001 | -0.026 | 0.113 |
| green | 70 | Kcnq1ot1 | 0.081 | 0.001 | -0.012 | 0.473 |
| turquoise | 283 | Ptprm | 0.053 | 0.001 | -0.017 | 0.269 |
| purple | 29 | Egfem1 | -0.052 | 0.001 | -0.021 | 0.189 |
| yellow | 78 | Slit2 | -0.038 | 0.023 | -0.028 | 0.077 |
| brown | 146 | Laptm5 | -0.005 | 0.761 | -0.004 | 0.766 |
| pink | 52 | Aldoc | -0.002 | 0.892 | 0.004 | 0.778 |

Spearman ρ of each of the ten module eigengenes versus mobility, before and after within-type centering. Unadjusted: n = 3,906 cells with matched RNA and t-type. Residual: n = 3,902 cells in types with ≥3 members (a subset of the 3,906). Empirical p from 1,000 label shuffles. Related to Figure 4; the same contrasts are plotted in Extended Data Figure 4-4B.
