# Supplementary material for: Flexible Cellular Phenotypes Retain a Shared Type-Level Organization across Response Regimes
Source: eNeuro. 2026 Nov 2;13(11):ENEURO.0275-26.2026. doi: 10.1523/ENEURO.0275-26.2026 (PMC13737997; doi:10.1523/ENEURO.0275-26.2026)
Supplement: Supplementary file 5 [file eneuro-13-ENEURO.0275-26.2026-s013.docx]

**Extended Data Table 1-1. Features by protocol.**

| Response regime | IPFX feature | Source column |
| --- | --- | --- |
| Long | vrest | feat.long_squares.vrest |
| Long | ri | feat.long_squares.ri |
| Long | sag | feat.long_squares.sag |
| Long | tau | feat.long_squares.tau |
| Long | vm for sag | feat.long_squares.vm_for_sag |
| Long | f i curve slope | feat.long_squares.f_i_curve_slope |
| Long | adaptation | feat.long_squares.adaptation |
| Long | latency | feat.long_squares.latency |
| Long | avg isi | feat.long_squares.avg_isi |
| Long | upstroke downstroke ratio | feat.long_squares.upstroke_downstroke_ratio |
| Long | peak v | feat.long_squares.peak_v |
| Long | peak t | feat.long_squares.peak_t |
| Long | trough v | feat.long_squares.trough_v |
| Long | trough t | feat.long_squares.trough_t |
| Long | fast trough v | feat.long_squares.fast_trough_v |
| Long | fast trough t | feat.long_squares.fast_trough_t |
| Long | threshold v | feat.long_squares.threshold_v |
| Long | threshold i | feat.long_squares.threshold_i |
| Long | threshold t | feat.long_squares.threshold_t |
| Long | v baseline | feat.long_squares.v_baseline |
| Long | rheobase i | feat.long_squares.rheobase_i |
| Long | rheobase sweep.avg rate | feat.long_squares.rheobase_sweep.avg_rate |
| Long | rheobase sweep.stim amp | feat.long_squares.rheobase_sweep.stim_amp |
| Long | rheobase sweep.v baseline | feat.long_squares.rheobase_sweep.v_baseline |
| Long | rheobase sweep.sag | feat.long_squares.rheobase_sweep.sag |
| Long | rheobase sweep.latency | feat.long_squares.rheobase_sweep.latency |
| Long | rheobase sweep.index | feat.long_squares.rheobase_sweep.index |
| Long | rheobase sweep.sweep number | feat.long_squares.rheobase_sweep.sweep_number |
| Long | fi fit slope | feat.long_squares.fi_fit_slope |
| Long | hero sweep.avg rate | feat.long_squares.hero_sweep.avg_rate |
| Long | hero sweep.stim amp | feat.long_squares.hero_sweep.stim_amp |
| Long | hero sweep.v baseline | feat.long_squares.hero_sweep.v_baseline |
| Long | hero sweep.sag | feat.long_squares.hero_sweep.sag |
| Long | hero sweep.adapt | feat.long_squares.hero_sweep.adapt |
| Long | hero sweep.latency | feat.long_squares.hero_sweep.latency |
| Long | hero sweep.isi cv | feat.long_squares.hero_sweep.isi_cv |
| Long | hero sweep.mean isi | feat.long_squares.hero_sweep.mean_isi |
| Long | hero sweep.median isi | feat.long_squares.hero_sweep.median_isi |
| Long | hero sweep.first isi | feat.long_squares.hero_sweep.first_isi |
| Long | hero sweep.index | feat.long_squares.hero_sweep.index |
| Long | hero sweep.sweep number | feat.long_squares.hero_sweep.sweep_number |
| Long | input resistance | feat.long_squares.input_resistance |
| Ramp | ramps.upstroke downstroke ratio | feat.ramps.upstroke_downstroke_ratio |
| Ramp | ramps.peak v | feat.ramps.peak_v |
| Ramp | ramps.peak t | feat.ramps.peak_t |
| Ramp | ramps.trough v | feat.ramps.trough_v |
| Ramp | ramps.trough t | feat.ramps.trough_t |
| Ramp | ramps.fast trough v | feat.ramps.fast_trough_v |
| Ramp | ramps.fast trough t | feat.ramps.fast_trough_t |
| Ramp | ramps.threshold v | feat.ramps.threshold_v |
| Ramp | ramps.threshold i | feat.ramps.threshold_i |
| Ramp | ramps.threshold t | feat.ramps.threshold_t |
| Ramp | ramps.mean spike 0.upstroke downstroke ratio | feat.ramps.mean_spike_0.upstroke_downstroke_ratio |
| Ramp | ramps.mean spike 0.peak v | feat.ramps.mean_spike_0.peak_v |
| Ramp | ramps.mean spike 0.peak t | feat.ramps.mean_spike_0.peak_t |
| Ramp | ramps.mean spike 0.trough v | feat.ramps.mean_spike_0.trough_v |
| Ramp | ramps.mean spike 0.trough t | feat.ramps.mean_spike_0.trough_t |
| Ramp | ramps.mean spike 0.fast trough v | feat.ramps.mean_spike_0.fast_trough_v |
| Ramp | ramps.mean spike 0.fast trough t | feat.ramps.mean_spike_0.fast_trough_t |
| Ramp | ramps.mean spike 0.threshold v | feat.ramps.mean_spike_0.threshold_v |
| Ramp | ramps.mean spike 0.threshold i | feat.ramps.mean_spike_0.threshold_i |
| Ramp | ramps.mean spike 0.threshold t | feat.ramps.mean_spike_0.threshold_t |
| Brief | upstroke downstroke ratio | feat.short_squares.upstroke_downstroke_ratio |
| Brief | peak v | feat.short_squares.peak_v |
| Brief | peak t | feat.short_squares.peak_t |
| Brief | trough v | feat.short_squares.trough_v |
| Brief | trough t | feat.short_squares.trough_t |
| Brief | fast trough v | feat.short_squares.fast_trough_v |
| Brief | fast trough t | feat.short_squares.fast_trough_t |
| Brief | threshold v | feat.short_squares.threshold_v |
| Brief | threshold i | feat.short_squares.threshold_i |
| Brief | threshold t | feat.short_squares.threshold_t |
| Brief | stimulus amplitude | feat.short_squares.stimulus_amplitude |
| Brief | mean spike 0.upstroke downstroke ratio | feat.short_squares.mean_spike_0.upstroke_downstroke_ratio |
| Brief | mean spike 0.peak v | feat.short_squares.mean_spike_0.peak_v |
| Brief | mean spike 0.peak t | feat.short_squares.mean_spike_0.peak_t |
| Brief | mean spike 0.trough v | feat.short_squares.mean_spike_0.trough_v |
| Brief | mean spike 0.trough t | feat.short_squares.mean_spike_0.trough_t |
| Brief | mean spike 0.fast trough v | feat.short_squares.mean_spike_0.fast_trough_v |
| Brief | mean spike 0.fast trough t | feat.short_squares.mean_spike_0.fast_trough_t |
| Brief | mean spike 0.threshold v | feat.short_squares.mean_spike_0.threshold_v |
| Brief | mean spike 0.threshold i | feat.short_squares.mean_spike_0.threshold_i |
| Brief | mean spike 0.threshold t | feat.short_squares.mean_spike_0.threshold_t |

IPFX feature column names retained for long-pulse, ramp, and brief-pulse phenotype construction. Related to Table 1 and Extended Data Figure 1-1.
