# Supplementary material for: Flexible Cellular Phenotypes Retain a Shared Type-Level Organization across Response Regimes
Source: eNeuro. 2026 Nov 2;13(11):ENEURO.0275-26.2026. doi: 10.1523/ENEURO.0275-26.2026 (PMC13737997; doi:10.1523/ENEURO.0275-26.2026)
Supplement: Supplementary file 6 [file eneuro-13-ENEURO.0275-26.2026-s014.docx]

**Extended Data Table 2-1. Statistical inventory.**

| Question | Test | Contrast | n | Effect size | Null mean | Empirical p | Notes |
| --- | --- | --- | --- | --- | --- | --- | --- |
| Cross-regime phenotype (Fig. 1) | Spearman of cell–cell D | Long–Ramp | 4054 | 0.434 | 0.000 | 0.0099 | 15-NN Jaccard=0.0228 |
| Cross-regime phenotype (Fig. 1) | Spearman of cell–cell D | Long–Brief | 4336 | 0.336 | 0.000 | 0.0099 | 15-NN Jaccard=0.0225 |
| Cross-regime phenotype (Fig. 1) | Spearman of cell–cell D | Ramp–Brief | 4134 | 0.384 | 0.000 | 0.0099 | 15-NN Jaccard=0.0185 |
| Transcriptomic anchoring (Fig. 2) | Distance alignment Aₜ (t-type) | Long | 4183 | 0.021 | -0.005 | 0.0099 | k=11 |
| Transcriptomic anchoring (Fig. 2) | Distance alignment Aₚ (Cre) | Long | 4183 | 0.069 | n/a | 0.0099 | k=11 |
| Transcriptomic anchoring (Fig. 2) | Distance alignment Aₚ (hierarchical subtype) | Long | 4183 | 0.212 | n/a | 0.0099 | k=11 |
| Transcriptomic anchoring (Fig. 2) | Distance alignment Aₜ (t-type after Cre) | Long | 4183 | 0.011 | n/a | 0.0099 | k=11 |
| Transcriptomic anchoring (Fig. 2) | η² (t-type) | Long | 4183 | 0.327 | n/a | n/a | k=11; descriptive (no shuffle p) |
| Transcriptomic anchoring (Fig. 2) | η² (Cre) | Long | 4183 | 0.209 | n/a | n/a | k=11; descriptive (no shuffle p) |
| Transcriptomic anchoring (Fig. 2) | η² (t-type after Cre) | Long | 4183 | 0.110 | n/a | n/a | k=11; descriptive (no shuffle p) |
| Transcriptomic anchoring (Fig. 2) | Same-type kNN enrichment | Long | 4183 | 6.590 | n/a | n/a | k=11; descriptive (no shuffle p) |
| Transcriptomic anchoring (Fig. 2) | Distance alignment Aₜ (t-type) | Ramp | 3988 | 0.025 | -0.002 | 0.0099 | k=3 |
| Transcriptomic anchoring (Fig. 2) | Distance alignment Aₚ (Cre) | Ramp | 3988 | 0.055 | n/a | 0.0099 | k=3 |
| Transcriptomic anchoring (Fig. 2) | Distance alignment Aₚ (hierarchical subtype) | Ramp | 3988 | 0.173 | n/a | 0.0099 | k=3 |
| Transcriptomic anchoring (Fig. 2) | Distance alignment Aₜ (t-type after Cre) | Ramp | 3988 | 0.010 | n/a | 0.0099 | k=3 |
| Transcriptomic anchoring (Fig. 2) | η² (t-type) | Ramp | 3988 | 0.393 | n/a | n/a | k=3; descriptive (no shuffle p) |
| Transcriptomic anchoring (Fig. 2) | η² (Cre) | Ramp | 3988 | 0.267 | n/a | n/a | k=3; descriptive (no shuffle p) |
| Transcriptomic anchoring (Fig. 2) | η² (t-type after Cre) | Ramp | 3988 | 0.120 | n/a | n/a | k=3; descriptive (no shuffle p) |
| Transcriptomic anchoring (Fig. 2) | Same-type kNN enrichment | Ramp | 3988 | 3.445 | n/a | n/a | k=3; descriptive (no shuffle p) |
| Transcriptomic anchoring (Fig. 2) | Distance alignment Aₜ (t-type) | Brief | 4266 | 0.021 | 0.005 | 0.0099 | k=5 |
| Transcriptomic anchoring (Fig. 2) | Distance alignment Aₚ (Cre) | Brief | 4266 | 0.037 | n/a | 0.0099 | k=5 |
| Transcriptomic anchoring (Fig. 2) | Distance alignment Aₚ (hierarchical subtype) | Brief | 4266 | 0.127 | n/a | 0.0099 | k=5 |
| Transcriptomic anchoring (Fig. 2) | Distance alignment Aₜ (t-type after Cre) | Brief | 4266 | 0.004 | n/a | 0.0099 | k=5 |
| Transcriptomic anchoring (Fig. 2) | η² (t-type) | Brief | 4266 | 0.149 | n/a | n/a | k=5; descriptive (no shuffle p) |
| Transcriptomic anchoring (Fig. 2) | η² (Cre) | Brief | 4266 | 0.110 | n/a | n/a | k=5; descriptive (no shuffle p) |
| Transcriptomic anchoring (Fig. 2) | η² (t-type after Cre) | Brief | 4266 | 0.040 | n/a | n/a | k=5; descriptive (no shuffle p) |
| Transcriptomic anchoring (Fig. 2) | Same-type kNN enrichment | Brief | 4266 | 2.643 | n/a | n/a | k=5; descriptive (no shuffle p) |
| Type–type S maps (Fig. 3) | Spearman of type–type S | Long–Ramp | 1596 | 0.658 | -0.003 | 0.000999 | n types=57; 1000 type-label shuffles; Table 2 j |
| Type–type S maps (Fig. 3) | Spearman of type–type S | Long–Brief | 1596 | 0.455 | -0.012 | 0.000999 | n types=57; 1000 type-label shuffles; Table 2 l |
| Type–type S maps (Fig. 3) | Spearman of type–type S | Ramp–Brief | 1596 | 0.608 | -0.016 | 0.000999 | n types=57; 1000 type-label shuffles; Table 2 k |
| Type–type S maps (Fig. 3) | Pair-tag counts (descriptive) | All eligible type pairs | 1596 | stable 639; intermediate 801; selective 156 | n/a | n/a | stable if rank-CV < 0.25; selective if rank-range ≥ 0.55 |
| RNA distance T (Fig. 4) | Spearman ρ(T, mean S) | Curated (263 genes) | 1596 | 0.551 | n/a | 0.0196 | n genes=263 |
| RNA distance T (Fig. 4) | Spearman ρ(T, rank-range) | Curated (263 genes) | 1596 | 0.011 | n/a | 0.7059 | n genes=263 |
| RNA distance T (Fig. 4) | Spearman ρ(T, mean S) | Primary (500 genes) | 1596 | 0.507 | 0.0007 | 0.000999 | n genes=500; 1000 shuffles; Table 2 m |
| RNA distance T (Fig. 4) | Spearman ρ(T, rank-range) | Primary (500 genes) | 1596 | 0.083 | 0.0009 | 0.000999 | n genes=500; 1000 shuffles; Table 2 n |
| RNA distance T (Fig. 4) | Spearman ρ(T, mean S) | HVG500 | 1596 | 0.520 | n/a | 0.0196 | n genes=500 |
| RNA distance T (Fig. 4) | Spearman ρ(T, rank-range) | HVG500 | 1596 | 0.105 | n/a | 0.0196 | n genes=500 |
| Excitability / marker modules (Fig. 4) | Spearman of module distance vs mean S | Nav (excitability) | 9 | 0.183 | n/a | 0.0196 | ρ vs rank-range=0.0429 |
| Excitability / marker modules (Fig. 4) | Spearman of module distance vs mean S | Kv3 (excitability) | 4 | 0.410 | n/a | 0.0196 | ρ vs rank-range=-0.0050 |
| Excitability / marker modules (Fig. 4) | Spearman of module distance vs mean S | Kv_delayed (excitability) | 7 | 0.086 | n/a | 0.0196 | ρ vs rank-range=-0.0408 |
| Excitability / marker modules (Fig. 4) | Spearman of module distance vs mean S | HCN (excitability) | 4 | 0.283 | n/a | 0.0196 | ρ vs rank-range=-0.0398 |
| Excitability / marker modules (Fig. 4) | Spearman of module distance vs mean S | Cav (excitability) | 8 | 0.021 | n/a | 0.4314 | ρ vs rank-range=0.0527 |
| Excitability / marker modules (Fig. 4) | Spearman of module distance vs mean S | SK_BK (excitability) | 7 | -0.055 | n/a | 0.0784 | ρ vs rank-range=-0.0394 |
| Excitability / marker modules (Fig. 4) | Spearman of module distance vs mean S | K_leak (excitability) | 7 | 0.238 | n/a | 0.0196 | ρ vs rank-range=-0.0227 |
| Excitability / marker modules (Fig. 4) | Spearman of module distance vs mean S | GABA_A (excitability) | 11 | -0.014 | n/a | 0.5098 | ρ vs rank-range=-0.0061 |
| Excitability / marker modules (Fig. 4) | Spearman of module distance vs mean S | AMPA_NMDA (excitability) | 9 | 0.028 | n/a | 0.3529 | ρ vs rank-range=0.0413 |
| Excitability / marker modules (Fig. 4) | Spearman of module distance vs mean S | Pvalb_marker (marker control) | 2 | 0.543 | n/a | 0.0196 | ρ vs rank-range=-0.0797 |
| Excitability / marker modules (Fig. 4) | Spearman of module distance vs mean S | Sst_marker (marker control) | 4 | 0.047 | n/a | 0.0980 | ρ vs rank-range=0.1476 |
| Excitability / marker modules (Fig. 4) | Spearman of module distance vs mean S | Vip_marker (marker control) | 3 | 0.300 | n/a | 0.0196 | ρ vs rank-range=0.0241 |
| Split-half neighborhood (Fig. 1-3) | Mean 15-NN Jaccard | Long-square half-A vs half-B | 3598 | 0.109 | 0.0026 | n/a | obs=0.109; chance=0.0026; adjusted=0.107; Table 2 u; 100 index shuffles |
| Split-half neighborhood (Fig. 1-3) | Mean 15-NN Jaccard (cross-regime) | Long–Ramp / Long–Brief / Ramp–Brief within split-half-eligible pool | 3276 / 3509 / 3351 | 0.025 / 0.024 / 0.020 | n/a | n/a | Cross-regime kNN recomputed within cells from the 3598-cell split-half pool; Table 2 v |
| Neighborhood-size sweep (Fig. 1-2) | Chance-adjusted kNN Jaccard at k=600 | Long-Ramp / Long-Brief / Ramp-Brief | 4054 / 4336 / 4134 | 0.134 / 0.101 / 0.108 | n/a | n/a | observed - chance; 100 index shuffles; Table 2 w |
| Cell-level mobility (Fig. 4-4) | 1 - mean Spearman of rank-distance profiles | Triple-shared cells | 4049 | 0.589 | n/a | n/a | sd=0.110; Table 2 x |
| WGCNA module vs mobility (Fig. 4-3; Fig. 4-4) | Spearman ρ after t-type residual | All 10 modules | 3902 | \|ρ\| ≤ 0.039 | n/a | n/a | no module with \|ρ\|≥0.10 and emp p<0.05; 1000 label shuffles; Table 2 y |

Master list of reported contrasts. Expands on Table 2. Related to Table 1, Table 2, and Figures 1–4.
